# *CsAP3*: A Cucumber Homolog to *Arabidopsis APETALA3* with Novel Characteristics

**DOI:** 10.3389/fpls.2016.01181

**Published:** 2016-08-04

**Authors:** Jin-Jing Sun, Feng Li, Dong-Hui Wang, Xiao-Feng Liu, Xia Li, Na Liu, Hai-Tao Gu, Cheng Zou, Jing-Chu Luo, Chao-Xing He, San-Wen Huang, Xiao-Lan Zhang, Zhi-Hong Xu, Shu-Nong Bai

**Affiliations:** ^1^State Key Laboratory of Protein and Plant Gene ResearchBeijing, China; ^2^Institute of Vegetables and Flowers, Chinese Academy of Agricultural SciencesBeijing, China; ^3^College of Life Sciences, Peking UniversityBeijing, China; ^4^Department of Vegetable Sciences, Beijing Key Laboratory of Growth and Developmental Regulation for Protected Vegetable Crops, China Agricultural UniversityBeijing, China; ^5^National Center of Plant Gene ResearchBeijing, China

**Keywords:** cucumber, unisexual flower development, MADS box genes, ethylene, stamen arrestment

## Abstract

In our previous efforts to understand the regulatory mechanisms of cucumber unisexual flower development, we observed a stamen-specific down-regulation of the ethylene receptor *CsETR1* in stage 6 female flowers of cucumber (*Cucumis sativus* L.). This down-regulation is correlated with the primordial anther-specific DNA damage that characterizes inappropriate stamen development in cucumber female flowers. To understand how *CsETR1* is down regulated in the stamen, we characterized a cucumber MADS box gene homologous to *Arabidopsis AP3*, *CsAP3*. We demonstrated that *CsAP3* is functionally equivalent to the *Arabidopsis* B-class MADS gene *AP3*. However, three novel characteristics of CsAP3 were found. These include firstly, binding and activating *CsETR1* promoter *in vitro* and *in vivo*; secondly, containing a GV repeat in its C-terminus, which is conserved in cucurbits and required for the transcription activation; and thirdly, decreased expression as the node number increases, which is similar to that found for *CsETR1*. These findings revealed not only the conserved function of *CsAP3* as a B-class floral identity gene, but also its unique functions in regulation of female flower development in cucumber.

## Introduction

Unisexual flowers are an efficient way to promote outcrossing and increase the genetic diversity of flowering plants (Bai and Xu, 2013). Understanding the mechanism of unisexual flower development is also important in crop improvement, especially for the crops and vegetables bearing unisexual flowers, such as maize (*Zea mays* L.) and cucumber (*Cucumis sativus* L.). For these plants, effective ways of regulating unisexual flower development could increase the efficiency of seed and fruit production.

Cucumber is a well-established model system for unisexual flower development (Perl-Treves, 1999; Bai and Xu, 2013). Since the late 1950’s, two approaches have been taken to decipher the regulatory mechanism of unisexual flower development. The physiological approach led to Witter and Bokovac (1958) reporting that ethylene plays key roles in promoting female flowers. Based on genetic approaches, it was proposed that interactions of three Mendelian genes, *F*, *M*, and *A*, determine whether a bisexual floral bud develops into a male or female flower (Galun, 1961; Kubicki, 1969a,b; Perl-Treves, 1999). Recently, all three genes have been cloned and found to encode 1-aminocyclopropane-1-carboxylic acid synthase, the rate-limiting enzyme in ethylene biosynthesis. *F* encodes *CsACS1G*, *M* encodes *CsACS2*, and *A* encodes *CsACS11* (Kamachi et al., 1997; Trebitsh et al., 1997; Mibus and Tatlioglu, 2004; Boualem et al., 2008, 2009, 2015; Li et al., 2009; Zhang et al., 2015). This progress confirms at the molecular level that ethylene plays a key role in unisexual flower development in cucumber, consistent with the correlation of ethylene production and genotypes of cucumber unisexual flowers reported by Yamasaki et al. (2001). However, little is known about how these *ACS* genes affect unisexual flower development.

To understand how a bisexual floral bud develops into either male or female flower, we systematically analyzed the morphogenetic process of cucumber unisexual flowers and revealed that the morphological divergence started from stage 6 flowers (Bai et al., 2004). We further found that the earliest observable biochemical difference is DNA damage at stamen primordia of stage 7 female flowers, which correlated with the arrest of stamen development (Hao et al., 2003). Since physiological investigations have demonstrated the effects of ethylene in promoting female flowers, we investigated whether the DNA damage at the arrested stamens is induced by ethylene. After demonstrating ethylene-induced DNA damage in cucumber protoplasts, we found that the expression of an ethylene receptor gene *CsETR1* is stamen-specifically down regulated in stage 6 female floral buds (Wang et al., 2010). Considering the ethylene receptors act as negative regulators of ethylene signal (Hua and Meyerowitz, 1998), stamen-specific down-regulation of the ethylene receptor gene *CsETR1* may increase the sensitivity of the stamen to ethylene. Together with the finding of preferential function of an ethylene-inducible DNase gene *CsCaN* in the stamen primordia of female floral buds (Gu et al., 2011), we proposed that down-regulation of *CsETR1* induces stamen-specific DNA damage, leading to an arrest in stamen development in female flowers (Bai and Xu, 2012, 2013). However, it is unknown how *CsETR1* is specifically down regulated in the stamens of cucumber female flowers.

One of the most significant breakthroughs in plant developmental biology in the past three decades is the identification of genes determining the developmental identity of floral organs, summarized as the “ABC model” (Coen and Meyerowitz, 1991; Weigel and Meyerowitz, 1994). These findings suggested that floral organ identities are determined by interactions of a group of MADS box genes, i.e., “A” class genes (*AP2* in *Arabidopsis* and their homologs in other plants) alone specify sepal identity; the combination of “A” and “B” class genes (*AP3*/*PI* in *Arabidopsis*, *GLO*/*DEF* in Antirrhinum and their homologs in other plants) specifies petal identity; the combination of “B” and “C” class genes (*AG* in *Arabidopsis*, *PLE* in *Antirrhinum* and their homologs in other plants) specifies stamen identity; and “C” class genes alone specify carpel identity (Bowman et al., 1989, 1991; Carpenter and Coen, 1990; Schwarz-Sommer et al., 1990; Sommer et al., 1990; Yanofsky et al., 1990; Jack et al., 1992; Goto and Meyerowitz, 1994). From the perspective of the “ABC model”, it is reasonable to expect that the stamen-specific down-regulation of *CsETR1* in cucumber female flowers could be mediated by a cucumber homolog of the B or C class genes that are responsible for stamen organ identity determination in other species.

To explore whether B or C class genes are involved in the stamen-specific down-regulation of *CsETR1* expression, detailed functional analyses of these genes are required. When we started this investigation, one B class gene C*UM26* and two C class genes, namely *CUM1* and *CUM10*, were reported in cucumber (Kater et al., 1998, 2001). None of them seems involved in stamen-specific DNA damage and arrest. As no cucumber genome was available until Huang et al. (2009), to identify candidate genes involved in down-regulating *CsETR1* expression in the stamens of female flowers, we performed suppression subtractive hybridization (SSH). We identified a MADS box gene of unknown function named *CsMADS1* that was differentially expressed in stamens of stage 5 and stage 6 female flowers (Gu et al., 2011).

In this work, we analyzed the characteristics of *CsMADS1* in detail. Based on sequence similarity, expression patterns and genetic complementary tests, we demonstrated that this MADS box gene encodes a homolog of *Arabidopsis* AP3 and therefore renamed it as *CsAP3* for clarity. In addition, we found that CsAP3 can bind and activate the promoter of *CsETR1*, that this activation ability required a GV repeat in its C-terminus, and that *CsAP3* expression is decreased in the flowers at higher nodes, consistent with the expression pattern of *CsETR1* (Wang et al., 2010). These findings revealed that while the CsAP3 has an equivalent function in organ identity determination to *Arabidopsis* AP3, it has novel characteristics required for the stamen-specific regulation of *CsETR1* expression. These novel characteristics provided a new example for functional divergence of ABC genes.

## Materials and Methods

### Plant Materials and Growth Conditions

Seeds of the monoecious cucumber (*Cucumis sativus* L.) line Zhongnong No.5 were purchased from the Institute of Vegetables and Flowers, Chinese Academy of Agricultural Sciences (CAASs). The seeds of hermaphrodite line H34 were kindly provided by Prof. Run Cai of Shanghai Jiaotong University. The seeds were sown in soil and plants were grown in a growth room under a day/night light regime of 16/8 h and a temperature of 25/18°C for sample collection.

Plants used for examination of gene expression in the floral buds at various nodes were grown in a greenhouse of the Institute of Vegetables and Flowers, CAAS. Two methods were used to collect floral samples at various nodes. Method 1 was as follows: when plants had 8-9 true leaves and floral buds of the 8th node were around stage 6–8 (when male and female buds could be easily distinguished by eye), floral buds of the 8th nodes and below were collected, separately for male and female, from five plants; floral buds 9–12 were collected from another five plants when the plants had 10–12 true leaves and the floral buds of 12th node were around stage 6–8; floral buds of 15th node and above contained in shoot tips were collected by simply collecting shoot tips of an additional five plants (Supplementary Figure S1A). Method 2 was as follows: five plants were grown to more than 15 nodes. Male and female floral buds around stage 6–8 were collected and grouped according to 8th node and below, 9–12th node, and 15th node and above (Supplementary Figure S1B).

*Arabidopsis* seeds were treated with 10% NaClO for 10 min and then washed by sterilized water for five times. The seeds were spread on the MS media. *Arabidopsis* plants were grown under long-day conditions (16-h-light/8-h-dark cycle) at 22°C and transgenic plants were generated using the floral-dip method (Clough and Bent, 1998).

### Construction of Transgenic Plants

The *ap3–13* mutant was kindly provided by Tom Jack of Dartmouth College. The *CsMADS1* complementation line was constructed by transforming the *ap3–13* heterozygous plants with *P_35S_:CsMADS1* plasmid (for detailed information see Supplementary Figure S2).

The dual transgenic *Arabidopsis* lines used for examining the activity of CsAP3 on the *CsETR1* promoter were constructed by transforming pER10 or pX6 vector containing CsAP3 into the transgenic *Arabidopsis* containing the *P_CsETR1_:GUS* construct (Supplementary Figure S3). After 14 days of selection on MS containing hygromycin B and kanamycin surviving seedlings were transferred to the MS medium containing 17-β-estradiol. After an induction period of 48 h, RNA was extracted and real-time qPCR was performed to monitor the expression of *CsAP3* and *GUS*. The lines in which the expression of *CsAP3* can be induced were used to detect the GUS expression, and those in which the expression of *CsAP3* cannot be induced were used as negative controls (for detailed information see Supplementary Figure S4). The induction system was previously reported (Zuo et al., 2000, 2001). pER10 and pX6 vectors were provided by Nam-Hai Chua.

### Phylogenetic Analyses

The protein sequence of *Arabidopsis thaliana* AP3 was used to search for MADS homologs in available genome sequences of 11 representative species^1^^,^^2^^,^^3^ (Supplementary Table S1). Multiple sequence alignment was performed using ClustalX (Plate-Forme de Bio-Informatique, Illkirch Cedex, France), and phylogenetic analysis of the whole sequences was performed using neighbor-joining (NJ) with the complete deletion option, the Jones–Taylor–Thornton (JTT) model, Gamma Distribution (G) of 1, and 1000 bootstrap replicates in MEGA version 5 (Tamura et al., 2011)^4^.

### Real-Time qPCR Analyses

Total RNA was isolated with Trizol Reagent (Invitrogen #15596-026), digested with DNase I (Takara #2270A) and reverse transcribed to cDNA with Superscript-III reverse transcriptase (Invitrogen # 18080-044) according to the user manual. qPCR analyses were performed using SYBR Premix Ex Taq Mix (Takara # RR420A) and ABI7500 according to the manufacturer’s instructions. The relative expression was calculated according the comparative C_T_ methods (Schmittgen and Livak, 2008). *CsACTIN2* (*Csa6M484600*) and *CsTUB1* (*Csa4M000580*) for cucumber and *UBIQUITIN* (*AT4G05320*) for *Arabidopsis* were used as reference. Primers were designed using Primer Primier 5 and are listed in Supplementary Table S2. For each sample, at least three biological replicates were analyzed and for each PCR, at least three technical replicates were used to calculate the C_T_ value.

### *In situ* and Whole-Mount *In situ* Hybridization Analyses

Shoot tips containing flower buds at various stages were fixed in 4% paraformaldehyde (PFA) overnight at 4°C. After fixation, tissues were washed, dehydrated, and embedded in wax for sectioning and *in situ* hybridization as described (Zhang et al., 2013). Tissues for whole-mount *in situ* hybridization were not embedded, and after dehydration, the procedure was performed according to the methods previously reported (Hejatko et al., 2006). *CsAP3*-specific regions were amplified with corresponding primer pairs (see Supplementary Table S2) and transcribed *in vitro* as probes using the Digoxigenin RNA labeling kit (Roche # 11175025910).

### EMSA Assays

The full-length CDS regions of *CsAP3*, *CUM26*, *AP3*, and *PI* were cloned into pCold™ TF vector (TaKaRa #3365) and the verified constructs were transformed into BL21 (DE3) *E. coli* to express the corresponding proteins. IPTG was added into the LB medium when the OD_600_ of the BL21 cells was 0.4 to 0.6, and then the cultures were incubated at 16°C for 8 to 10 h. The proteins were purified with Ni-NTA Agarose (QIAGEN #30210) according to the user manual after the induction by IPTG. The purified proteins were analyzed by SDS-PAGE and quantified with the Bradford methods (Kruger, 1994). DNA fragments labeled with the Biotin 3′ End DNA Labeling Kit (Thermo #89818) were used as probes and unlabelled DNA fragments were used as competitor. CArG boxes were designed to be in the middle of the probes. EMSA assays were performed according to the instructions of the LightShift^®^ Chemiluminescent EMSA Kit (Thermo #20148). The probe sequences for EMSA are listed in Supplementary Table S2.

### ChIP-qPCR

ChIP was performed according to the protocols previously reported (Liu et al., 2013). The AP3 antibody, which recognizes a region conserved between CsAP3 and AP3, was purchased from Santa Cruz Biotechnology (sc-12639). Chromatin was extracted from flowers of cucumber and *Arabidopsis* and immunoprecipitated DNA fragments were used as templates for real-time qPCR with specific primers (See Supplementary Table S2). Leaf and IgG were used as negative controls. The same initial amounts of tissue and antibody were used for the leaf control and the same amounts of antibody and IgG was used for the IgG control. The internal reference for *Arabidopsis* was *TUBULIN2* (Liu et al., 2007). To find a proper internal reference for cucumber ChIP-qPCR, we examined the cucumber homolog of *Arabidopsis TUBULIN* and *ACTIN*, *CsTUBULIN*, and *CsACTIN*. After comparing the ChIP-qPCR results for relative fold enrichment of *CsTUBULIN* and *CsACTIN*, we found that they were almost the same. Based on these results, we used *CsTUBULIN* as the internal reference for cucumber.

### Nuclear and Cytoplasmic Protein Extraction

The indicated plant tissues were ground into fine powder with liquid nitrogen, pre-chilled mortars, and pestles. Then, the samples were resuspended in nuclear isolation buffer (Saleh et al., 2008) at 2 g tissue/25 mL buffer. After brief mixing, the homogenized slurry was filtered through double-layer MiraCloth (CALBIOCHEM #475855), then centrifuged at 11000 *g* for 20 min at 4°C. The nuclear material could be seen at the bottom of the tube as a white tight pellet overlaid by chlorophyll. The supernatants were saved as the cytoplasmic extract and cold nucleus lysis buffer (Saleh et al., 2008) was used to resuspend the pellet (nuclei). Nuclear protein was obtained from the extract by brief sonication and centrifugation at 13800 *g* for 20 min at 4°C.

### Transcription Activity Assay in Yeast

The cDNAs encoding the C-terminal regions of CsAP3, AP3, CsAP3_C_m (without the GV repeat) and AP3_C_m (with the GV repeat inserted into AP3) were cloned into pGBKT7 vectors and then transformed into *S. cerevisiae* strain AH109. Colonies were first screened on SD medium without Trp. Six independent positive clones were picked arbitrarily, and transferred to SD medium without Trp, His, Ade for high stringency selection. Vector construction, transformation procedures, and screening methods were according to Protocol No. PT3955-1 (Clontech Laboratories).

### Dual Luciferase Transient Expression Assay

The cDNAs encoding CsAP3, AP3, CsAP3_m (without the GV repeat) and AP3_m (with the GV repeat inserted into AP3) were cloned into the vector pGreenII-62SK. The 2,000 bp upstream of the *CsETR1* coding region were cloned into the vector pGreenII-0800-LUC (Hellens et al., 2005). The verified constructs were transformed into *Agrobacterium tumefaciens* strain GV3101 and the positive clones were incubated in LB medium until the OD600 was about 0.5. The cell culture was collected by centrifugation and re-suspended in IFB buffer (10 mM MES, 10 mM MgCl2, 150 μM acetosyringone), placed in the darkness for 3 h. The cell culture mixture containing the effector and reporter construct strains at a ratio of 9:1 was inoculated into tobacco (*Nicotiana benthamiana*) leaves. After about 48 h, leaf disks were collected, and the activity of firefly luciferase and *Renillia* luciferase were analyzed. Leaf disks inoculated with reporter and empty effector constructs were used as background controls. Firefly and *Renillia* luciferase assays were performed according to the instructions of the Dual-Luciferase^®^ Reporter Assay System (Promega #E1910) using a GloMaxR 20/20 Luminometer. For each sample, at least eight biological replicates were used.

## Results

### *CsAP3* is a Homolog of *Arabidopsis AP3*

**FIGURE 1:**
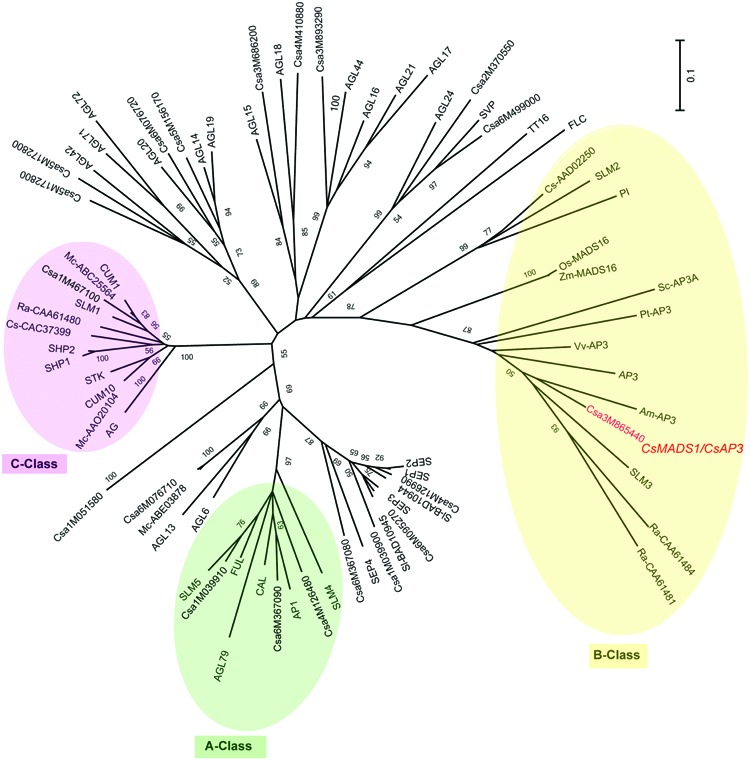
**Unrooted phylogenetic analysis of the MADS gene family**. An unrooted neighbor-joining (NJ) tree based on protein sequence data was constructed (see Materials and Methods). >50% bootstrap values are marked on the tree. A Class proteins are highlighted with a green circle; B Class proteins with a yellow circle; C Class proteins with a pink circle. Csa3M865440 is CsMADS1 (renamed herein CsAP3), and grouped with B Class protein AP3, but not PI. Am (*Antirrhinum majus*), At (*Arabidopsis thaliana*), Cs/Csa (*Cucumis sativus*), Mc (*Momordica charantia*), Os (*Oryza sativa*), Pt (*Populus trichocarpa*), Ra (*Rumex acetosa*), Sl (*Silene latifolia*), Sc (*Silene conica*), Vv (*Vitis vinifera*), Zm (*Zea mays*).

We previously identified a MADS box-containing EST with differential expression in stamens of various stages. Using 5′RACE (Rapid Amplification of cDNA Ends) and 3′RACE, we got the full length CDS and then deposited it to NCBI under the name of *CsMADS1* (accession number AY944060) (Gu et al., 2011). Later it was numerated as Csa3M865440 in the cucumber genome project (Huang et al., 2009). *CsMADS1* contains 732 bp encoding 244 amino acids. This sequence is highly similar to *Arabidopsis* AP3, with 58% identity at the amino acid level and contains all of the conserved domains found in typical MADS proteins (Supplementary Figure S5). Phylogenetic analysis with available information from sequenced organisms (Supplementary Table S1) grouped *CsMADS1* into the “B” class, sub-clustered with *Arabidopsis AP3*, and separate from cucumber *CUM26* (CsAAD02250) and *Arabidopsis PI* (**Figure 1**). Based on this information, we further analyzed the functions of this newly identified cucumber MADS box gene.

**FIGURE 2:**
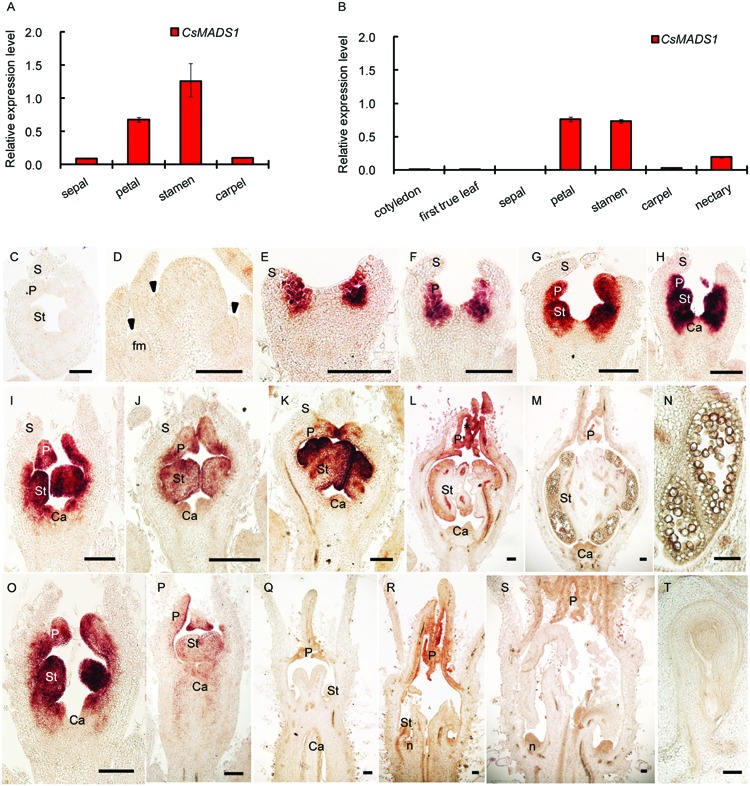
**Expression pattern of *CsMADS1/CsAP3.* (A–B)** Real-time qPCR analysis showing that *CsMADS1/CsAP3* expression was high in stamens and petals of hermaphrodite H34 floral buds at stage 6–8 (A) and monoecious female floral buds at stage 7–8 (B), but low in other organs. **(C–T)** Detection of *CsAP3* with in situ hybridization. **(C)** Flower bud hybridized with *CsAP3* sense probe. **(D–H)** Longitudinal sections of flower buds at stages 1–5. Arrowheads indicate newly initiated floral buds (early stage 1). **(I–M)** Longitudinal sections of stages 6–10 male flower buds. **(N)** Longitudinal section of a stamen of the stage 10 male flower bud. **(O–S)** *CsAP3* expression in female flower buds at stages 6, 7, 8–2, 8–3, and 9, respectively. **(T)** Longitudinal section of an ovule of a female bud at stage 11. fm, floral meristem; S, sepal; P, petal; St, stamen; Ca, carpel; n, nectary. Scale bars = 100 μm.

*CsMADS1* was preferentially expressed in the 2nd and 3rd whorls of floral organs (**Figures 2A,B,E–P**; Supplementary Figures S6A–K,M), similar to *AP3* in *Arabidopsis* (Jack et al., 1992). However, expression of *CsMADS1* was dramatically decreased from stage 7–8 in stamens of female flowers (**Figures 2P–R**; Supplementary Figure S6L), when the stamen development is clearly inhibited (Hao et al., 2003; Bai et al., 2004). The decreased expression in female flowers is different from *AP3* of *Arabidopsis* and from *CsMADS1* expression in the stamen of male cucumber flowers.

**FIGURE 3:**
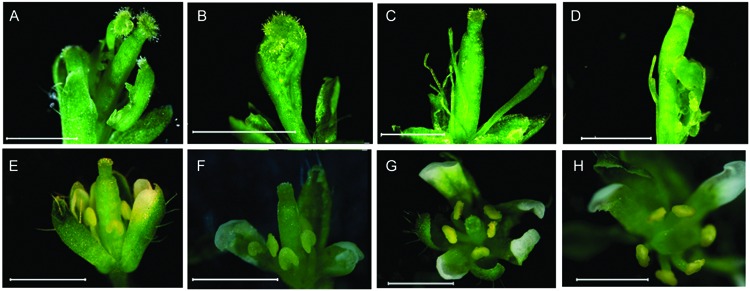
**Rescue of the *Arabidopsis ap3* phenotype with *CsMADS1.*** The *Arabidopsis ap3* mutant was transformed with a construct driving constitutive expression of *CsMADS1.* **(A–D)** Floral phenotypes of *ap3* homozygotes. **(E–H)** Floral phenotypes of *ap3* homozygotes were rescued with transgenic *CsMADS1* but with some remaining variations. **(E,F)** Independent lines 19 and 24. **(G,H)** Independent line 33.

To clarify whether *CsMADS1* is functionally equivalent to *AP3* in *Arabidopsis*, we carried out a genetic complementation test by transforming an *Arabidopsis ap3* mutant (*ap3–13*) with *CsMADS1* (Supplementary Figure S7). *CsMADS1* could successfully complement the *ap3* mutant phenotype (**Figure 3**), demonstrating that *CsMADS1* is functionally equivalent to *AP3* in terms of organ identity determination. Based on these results, we confirmed that CsMADS1 is the homolog of AP3 and re-designated *CsMADS1* as *CsAP3*.

### CsAP3 Binds the CArG Box in the *CsETR1* Promoter but AP3 Does Not Bind the *ETR1* CArG Box

**FIGURE 4:**
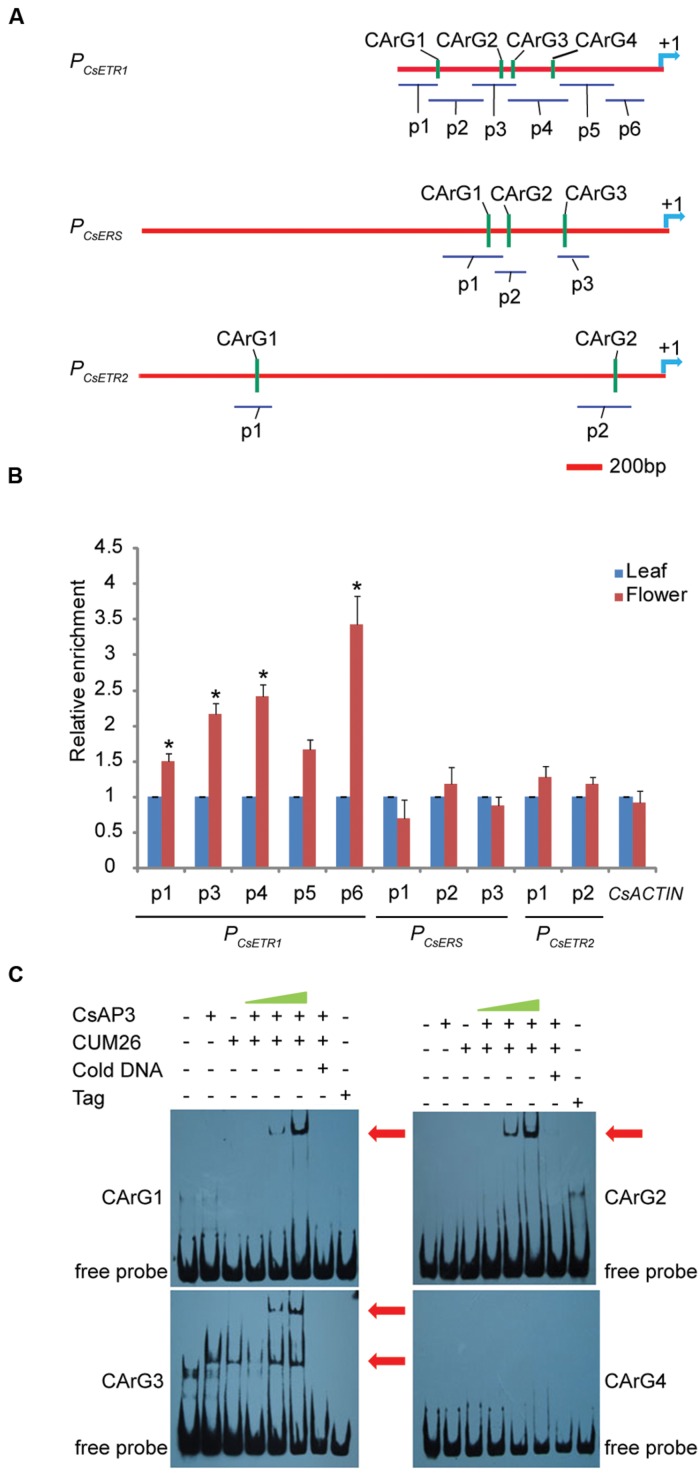
**CsAP3 can bind the *CsETR1* promoter *in vivo* and *in vitro***(A)** Diagram showing the positions of fragments used for examination of binding activity of CsAP3 to the *CsETR1* promoter *in vivo* by ChIP-qPCR (p1–p6) and *in vitro* by EMSA (CArG1–CArG4)**. Primers used in ChIP-qPCR for amplifying the fragments containing CArG box of *CsERS* and *CsETR2* promoters are also shown. The position +1 denotes the adenine of the translational start codon (ATG). **(B)** ChIP-qPCR showing that CsAP3 significantly enriched fragments of *CsETR1* promoter (p1–p4) that contain a CArG box but not p5 (p2 failed to amplify under various conditions). CsAP3 also significantly enriched fragment p6 although it contains no predicted CArG box. CsAP3 did not significantly enrich fragments containing CArG boxes of the *CsERS* or *CsETR2* promoter. The ChIP results are presented as enrichment relative to CsTUBULIN. Error bars indicate SD (*n* = 3). ^∗^*p* < 0.05 in Student’s *t*-test compared to negative control. **(C)** EMSA showing that CsAP3 directly bound fragments of the *CsETR1* promoter containing CArG boxes. Increasing concentrations of proteins were used and 500-fold molar excess unlabeled DNA fragments were added as competitor. The shifted bands are indicated with red arrowheads.

To examine whether CsAP3 is involved in the regulation of *CsETR1* transcription, we first analyzed the promoter of *CsETR1*, and found typical CArG boxes predicted to be MADS family protein binding sites (Schwarz-Sommer et al., 1992; Treisman and Ammerer, 1992; Riechmann et al., 1996) (Supplementary Table S3). Then, we carried out ChIP-qPCR assays using an antibody capable of recognizing CsAP3 protein (Supplementary Figures S8A,B) and found that the *CsETR1* promoter regions containing CArG boxes were significantly enriched in the flower samples but not in leaf samples, which showed no *CsAP3* gene expression (**Figures 4A,B**). When using IgG as another negative control, we got the similar results (Supplementary Figure S8C). These results indicated that CsAP3 binds the *CsETR1* promoter *in vivo*.

To examine whether CsAP3 can bind the promoters of two other ethylene receptor genes, we examined the distribution of CArG boxes on the promoters of *CsETR2* and *CsERS* and carried out ChIP-qPCR assays using the above antibody and primers covering these CArG boxes (**Figure 4A**). None of the examined *CsETR2* or *CsERS* promoter segments was enriched (**Figure 4B**; Supplementary Figure S8C). These results showed that CsAP3 can selectively bind the promoter of *CsETR1*, but not *CsETR2* or *CsERS*, suggesting that such binding might be significant in function.

To confirm the binding activity of CsAP3 to the *CsETR1* promoter, we further carried out Electrophoretic Mobility Shift Assays (EMSAs) with recombinant CsAP3 (Supplementary Figure S9A). Considering that AP3 functions coordinately with PI (Immink et al., 2010; Smaczniak et al., 2012b), CUM26 protein (Supplementary Figure S9A), the cucumber homolog of *Arabidopsis* PI (Kater et al., 2001), were used in the EMSA system together with CsAP3. Synthesized DNA fragments containing the CArG box of *CsETR1* promoter were used as probe as listed in Supplementary Figure S9B. CsAP3 interacted with CArG boxes 1-3, but not CArG box 4, in the presence of CUM26 (**Figure 4C**). These results demonstrated that CsAP3 proteins bind the *CsETR1* promoter directly.

**FIGURE 5:**
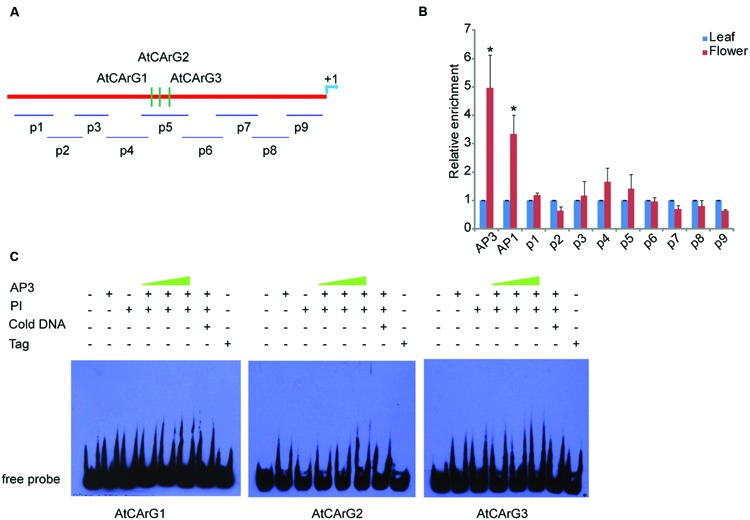
**AP3 cannot bind the *ETR1* promoter effectively *in vivo* or *in vitro* (A)** Diagram of the positions of fragments used for examination of binding activity of AP3 to the *ETR1* promoter [1448 bp upstream of ATG, according to Wang et al. (2003)] *in vivo* by ChIP-qPCR (p1–p9) and *in vitro* by EMSA (CArG1–CArG3). The position +1 denotes the adenine of the translational start codon (ATG). **(B)** ChIP-qPCR showing that AP3 did not enrich the fragments of the *ETR1* promoter. Confirmed binding sites of AP3 and AP1 (Sundstrom et al., 2006) were used as positive controls. The ChIP results are presented as relative enrichment to TUBULIN2. Error bars indicate SD (*n* = 3). ^∗^*p* < 0.05 and ^∗∗^*p* < 0.01, respectively, in Student’s *t*-test compared to negative control. **(C)** EMSA assays showed that AP3 cannot directly bind fragments of the *ETR1* promoter containing putative CArG boxes.

To clarify whether the binding of CsAP3 upon the *CsETR1* promoter is correlated with unisexual flower development in cucumber, we examined if *Arabidopsis* AP3 can bind *ETR1* promoter as *Arabidopsis* bears only perfect/bisexual flowers. We performed ChIP-qPCR assays with AP3 antibody and the *ETR1* promoter, which also contains CArG boxes (**Figure 5A**). The AP3 antibody could effectively enrich segments containing CArG boxes in *AP3* and *AP1* genes as previously reported (Sundstrom et al., 2006), demonstrating that the ChIP-qPCR assay worked in *Arabidopsis*. However, no CArG box-containing segments of the *ETR1* promoter were enriched by the AP3 antibody (**Figures 5A,B**). This result is consistent with recently published ChIP-seq data, in which no *ETR1*-related DNA sequence was enriched by AP3 antibody (Wuest et al., 2012). To further clarify the interaction between AP3 and CArG boxes in the *ETR1* promoter, we carried out EMSA. **Figure 5C** shows that CArG boxes were not bound by AP3 in the presence of PI *in vitro*. These results indicate that unlike the CsAP3, AP3 cannot bind the *ETR1* promoter, and imply that the CsAP3 binding to the *CsETR1* promoter may play roles in cucumber unisexual flower development.

### CsAP3 Contains an Additional C-terminal GV Repeat that is Required for Transcriptional Activation

**FIGURE 6:**
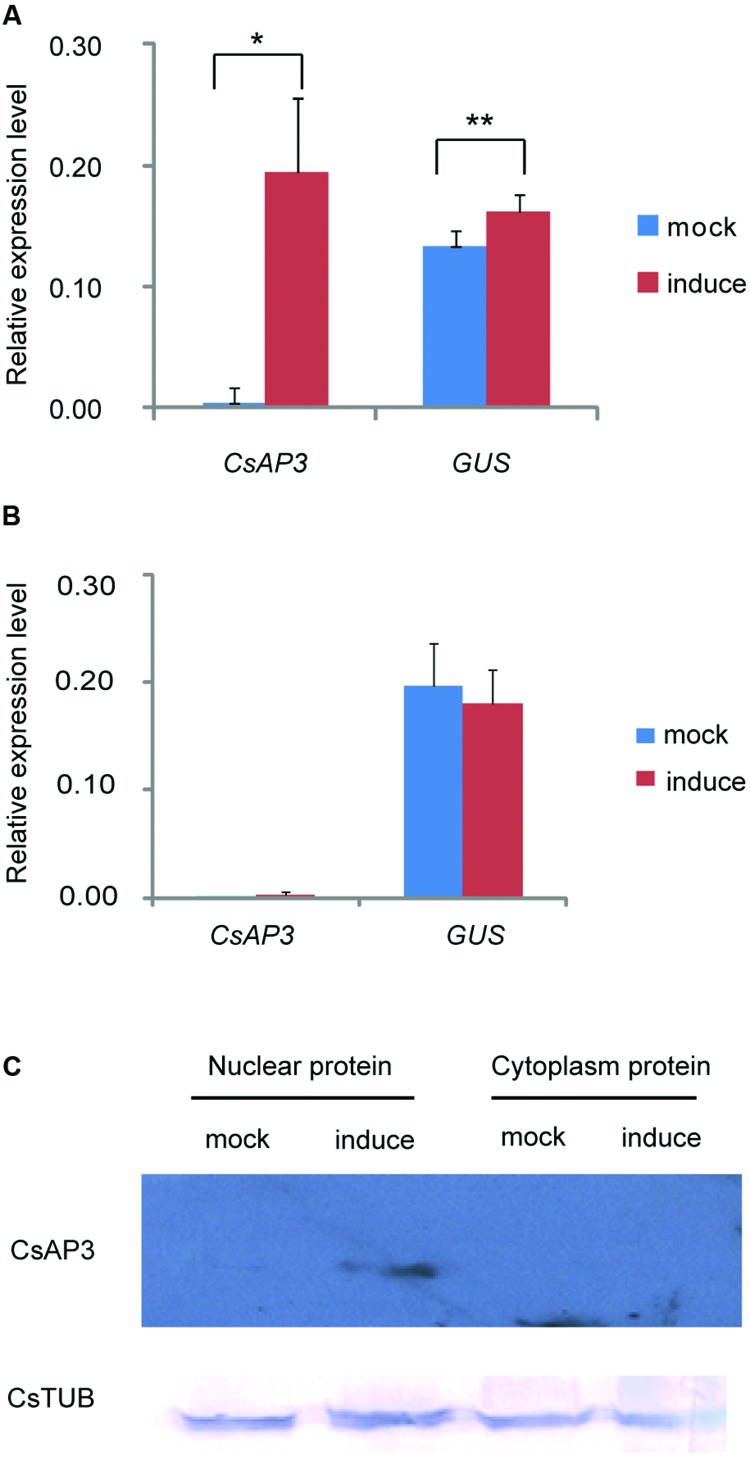
**CsAP3 can activate the *CsETR1* promoter in transgenic *Arabidopsis.* (A)** When expression of *CsAP3* was induced by the presence of estrogen, *GUS* expression was activated (red bar) to significantly higher levels than the background detected in the mock conditions, i.e., in the absence of estrogen (blue bars) **(B)** In the lines in which the expression of *CsAP3* could not be induced in the presence of estrogen, the *GUS* expression (red bar) was not activated above background levels (blue bar). **(C)** CsAP3 protein accumulated in the nuclear fraction upon application of estrogen, detected by immunoblotting with the AP3 antibody. CsTUB was used as the loading control, detected by immunoblotting with TUBULIN antibody. Data show mean ± SEM. *n* = 10 in **(A)** and *n* = 6 in **(B)**. ^∗^*p* < 0.05 and ^∗∗^*p* < 0.01, respectively, in Student’s *t*-test.

We asked whether CsAP3 binding to the *CsETR1* promoter could lead to transcriptional regulation of *CsETR1*. As there is currently no reliable protocol to routinely construct transgenic cucumber, we used transgenic *Arabidopsis* as an alternative. We constructed dual transgenic *Arabidopsis* lines containing a *CsETR1* promoter-driven *GUS* gene and an estrogen-inducible *G1090* promoter-driven *CsAP3* gene (derived from pER10 or pX6, Supplementary Figure S3). If CsAP3 were able to activate the *CsETR1* promoter, we expected to observe increased *GUS* expression upon estrogen-induced *CsAP3* expression in the double transgenic lines. Since not all the estrogen-treated plants resulted in induced *CsAP3* expression, we used those lines with no *CsAP3* induction as internal negative controls. In the lines in which the *CsAP3* mRNA was induced by estrogen and CsAP3 protein accumulated, *GUS* expression is indeed increased (**Figures 6A,C**). By contrast, in the lines in which the *CsAP3* was not induced, *GUS* expression did not change (**Figure 6B**). These data suggested that CsAP3 can activate the *CsETR1* promoter.

**FIGURE 7:**
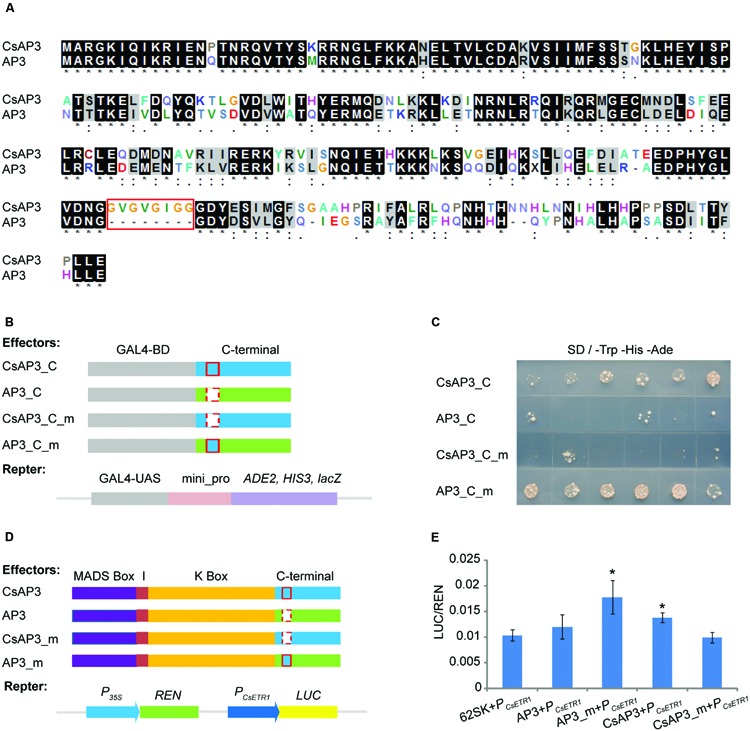
**The GV-repeat sequence confers transcriptional activation activity to the C-terminal regions of AP3 and CsAP3. (A)** ClustalX alignment of CsAP3 and AP3 protein sequences. Amino acid residues displaying 100% identity or similarity in the two homologs are shaded black and gray. The red rectangle shows the GV-repeat sequence in CsAP3. **(B)** Diagram of effector and reporter vector constructs for the transcriptional activity assay in yeast. Light blue and green show the C-terminal regions of CsAP3 and AP3, respectively. Red rectangles with solid and dashed lines indicate with and without the GV repeat, respectively. **(C)** Transcriptional activity assay in yeast. Yeast with CsAP3_C or AP3_C_m, both of which contain the GV repeat, could activate the reporter gene *ADE2* and *HIS3*. However, CsAP3_C_m or AP3_C, both lacking the GV repeat, could not. The spots from left to right are independent clones of the same type of transformant. **(D)** Diagram of effector and reporter vector constructs used in tobacco transient dual luciferase assays. Red rectangles with solid and dashed lines indicate with and without the GV repeat, respectively. **(E)** Dual luciferase transient expression assay in tobacco leaves. Compared to the negative control (62SK + P_CsETR1_), AP3_m and CsAP3 up-regulated the LUC/REN ratios significantly but AP3 and CsAP3_m did not. Error bars indicate SEM (*n* = 8). ^∗∗^*p* < 0.01, in Student’s *t*-test.

Based on the complementation of the *ap3* mutant phenotype, CsAP3 is functionally equivalent to *Arabidopsis* AP3 in stamen identity determination. However, it has additional functionality in binding and activating the *CsETR1* promoter. To clarify the reason, we carried out a detailed sequence comparison between the two proteins. While CsAP3 contains typical MADS and other domains, which are highly conserved and explain its functional equivalence as a B-class protein (Supplementary Figures S5), we found an additional 8-amino acid “GV repeat” (GVGVGIGG) from 185–192 aa of CsAP3 (**Figure 7A**, red framed). To clarify whether such a “GV repeat” might have functional significance despite occurring in the highly variable C-terminal region, we carried out a sequence comparison with available AP3 homologs from representative plant species. These plants included 6 cucurbits in addition to cucumber, namely *Momordica charantia*, *Luffa cylindrical*, *Cucurbita maxima*, *Cucurbita pepo*, *Cucumis melo*, *Citrullus lanatus*; and 7 non-cucurbits, in addition to *Arabidopsis*, *Antirrhinum majus*, *Nicotiana tabacum*, *Vitis vinifera*, *Euptelea pleiosperma*, *Akebia trifoliate*, *Oryza sativa*, and *Zea mays*. The GV repeat was identified in all cucurbits with little variation, but not in any of non-cucurbits even though two of these species, *Akebia trifloiata* and *Zea mays*, bear unisexual flowers (Supplementary Figure S10). It is known that all the examined cucurbits plants bear unisexual flowers and their female flowers are promoted by ethylene (Rudich, 1969; Byers et al., 1972; Hossain et al., 1995; Sugiyama et al., 1998; Manzano et al., 2009, 2014; Martinez et al., 2014). The sequence comparison strongly suggests that the GV repeat might have functional relevance to a common feature shared by the cucurbits.

To test whether the GV repeats in the C-terminal region, conserved in cucurbits, play any role in CsAP3 activation of the *CsETR1* promoter, we conducted transcription activity assays of the CsAP3 and AP3 C-terminus. **Figures 7B,C** shows that the C-terminus of CsAP3 (with the GV repeats) had transcriptional activity but AP3 (without the GV repeats) did not. Importantly, the C-terminus of CsAP3 lost the transcriptional activation activity when the GV repeats were deleted and the C-terminus of AP3 acquired transcriptional activation activity with the insertion of the GV repeats. These findings demonstrated that the GV repeat is required for transcriptional activity. This characteristic was confirmed in tobacco transient activation assays using full-length CsAP3, AP3 and their mutated forms, by adding the GV repeat to AP3 and removing it from CsAP3 respectively, with *CsETR1* promoter as a target (**Figures 7D,E**). The latter experiment not only verified the transcriptional activation activity of the GV repeat in the CsAP3 C-terminus, uncovering a functional difference between CsAP3 and AP3, but also strengthened the previous conclusion that CsAP3 activates the *CsETR1* promoter.

### The Expression of *CsAP3* Is Decreased at the Upper Nodes

The above results indicated that CsAP3 activates the *CsETR1* promoter, implying that higher *CsAP3* expression would lead to the higher *CsETR1* expression. This correlation of expression levels between *CsAP3* and *CsETR1* was indeed found in our examination of *CsAP3* expression (**Figure 2**) and the expression examination of *CsETR1* shown in Figure 4 of Wang et al. (2010). We found that *CsAP3* expression was higher at stage 8 in male flowers with a well-developed stamen, and lower in female flowers with the inhibited stamens (**Figure 2**). Consistent with these results, Wang et al. (2010) showed that the expression of *CsETR1* was lower in the stamens than that in the carpels of stage 6 female flowers and that in the stamens of stage 6 male flowers. This agreement in expression patterns supports the regulatory role of CsAP3 upon *CsETR1*.

**FIGURE 8:**
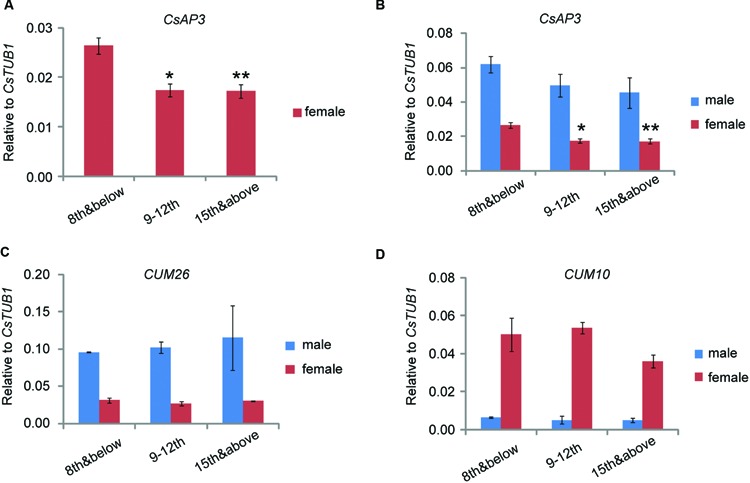
***CsAP3* expression decreases as the node number increases**. Expression levels of *CsAP3* **(A,B)**, *CUM26* **(C)**, and *CUM10* **(D)** in male and female floral buds collected at nodes 8 and below, nodes 9–12, and the shoot tip. The expression of *CsAP3* decreases in higher nodes, not only in male, but more significantly in female flowers **(A)**. The data in this figure are from buds collected from plants grown to the indicated number of nodes (Materials and Methods 1, Supplementary Figure S1A), but the trend of *CsAP3* expression in floral buds was independent of the method of sample collection (See Supplementary Figures S1B–E). Data are presented as mean ± SD (*n* = 3). ^∗^*p* < 0.05 and ^∗∗^*p* < 0.01, respectively, in Student’s *t*-test in comparison to the 8th and below value for the same sex floral buds. **(A)** is the magnified part of *CsAP3* expression in female flower from **(B)**.

We previously reported that *CsETR1* expression in stamen primordia of female floral buds decreased as the node number increased, consistent with the phenomenon that in monoecious cucumber, male flowers are produced in lower nodes and female flower are produced in higher nodes (Wang et al., 2010). If CsAP3 indeed plays role in regulating *CsETR1* expression in cucumber, *CsAP3* expression should also decrease in later nodes as that of *CsETR1* does. Accordingly, we examined *CsAP3* expression in stage 6–8 male and female floral buds collected at nodes 8 and below, nodes 9 to 12 and the shoot tip above node 15, with *CUM26* and *CUM10* expression used for comparison. To avoid interference from sampling methods, we collected samples in two different ways. In one way, we collected floral buds at stage 6-8 from plants grown to the designated nodes (Method 1, Supplementary Figure S1A). Using this method, *CsAP3* and *CUM26*, the two cucumber B class genes, were found to be expressed higher in male flowers than in female flowers (**Figure 8**). *CUM10*, the cucumber “C” class gene, was expressed more highly in female flowers than in male flowers (**Figure 8C**). These expression patterns of the three genes are in line with the respective organ development in unisexual flowers.

One phenomenon of particular interest was that although *CsAP3* had relatively lower expression in all female flowers, it exhibited a significant decrease in floral buds as the node number increased (**Figures 8A,B**). No such differences of *CUM26* and *CUM10* expression were observed in male or female flowers (**Figures 8C,D**). Similar results were obtained when we collected floral buds at stage 6–8 from the designated nodes in plants grown to more than 15 nodes (Method 2, Supplementary Figure S1B–E). Together with the lower expression of both *CsAP3* and *CsETR1* in the stamens of female compared to male flowers, the concordant decreases in expression of *CsAP3* and *CsETR1* in upper nodes suggests that CsAP3 might regulate *CsETR1* transcription.

## Discussion

To find potential regulators responsible for the stamen-specific down-regulation of *CsETR1* in female flowers of cucumber, we characterized the previously cloned *CsMADS1* gene that is preferentially expressed in stamen (Gu et al., 2011). We demonstrated that the *CsMADS1* gene is the *Arabidopsis AP3* homolog in cucumber and renamed it as *CsAP3*, based on analysis of sequence similarity, expression pattern and functional complementation. We further demonstrated that CsAP3 can bind CArG box-containing regions of the *CsETR1* promoter *in vitro* (based on EMSA) and *in vivo* (based on ChIP-qPCR), and that it activates the *CsETR1* promoter in the inducible assay system in transgenic *Arabidopsis* plants. Furthermore, we identified a C-terminal GV repeat in cucurbit AP3 homologs and showed that such a GV repeat is responsible for the ability of CsAP3 to transcriptionally activate *CsETR1*. Based on these findings together with the fact that expressions of both *CsAP3* and *CsETR1* decrease as the node numbers increase, we propose that CsAP3 likely plays a role in the regulation of the stamen-specific down-regulation of *CsETR1* in female cucumber flowers.

Although the lack of effective cucumber transformation methods prevented functional demonstration of the role of CsAP3 in regulating *CsETR1* transcription in planta, our work revealed two novel characteristics of CsAP3, in addition to the one that CsAP3 binds and activates the *CsETR1* promoter.

One such previously unknown characteristic is the GV repeat found in the C-terminus of CsAP3. B-class MADS box genes are required for stamen organ identity determination and are functionally conserved in various angiosperm species (Causier et al., 2010; Smaczniak et al., 2012a). There are four major domains in MADS box proteins (MADS, I, K, and C). The MADS box is a DNA-binding domain, whereas I and K are dimerization domains (Kaufmann et al., 2005; Smaczniak et al., 2012a). By contrast, the C domain is highly variable and little is known about its function. It has been reported that the C domain has transcriptional activation activity in AP1 and SEP3 but not in AP3, PI, and AG (Honma and Goto, 2001) and the C domain can enhance/stabilize interactions mediated by the K domain (Fan et al., 1997; Kaufmann et al., 2005; Immink et al., 2010). Others have reported that the C domain of AP3 and PI is dispensable for organ identity function (Piwarzyk et al., 2007; Benlloch et al., 2009; Smaczniak et al., 2012b). In database searches, we found the GV repeat in genes from various species including bacteria, fungi, animals and plants (Supplementary Table S4). Although simple sequence repeats in proteins have been described and investigated in various organisms (Urbanus et al., 2010; Dornelas et al., 2011), no functional information regarding the GV repeat was available before its role in transcriptional activation was discovered in this work. Since the GV repeat is highly conserved in cucurbits, it will be interesting to elucidate whether the GV repeat was co-opted exclusively into the AP3 homologs of cucumber relatives and how it occurred. This finding opens up new opportunities to understand the divergence of MADS box genes in sequences and related developmental functions during evolution, particularly in ethylene-related unisexual flower development.

The other novel characteristic discovered herein is the decreased expression of *CsAP3* with the node number increase. Although Schultz and Haughn (1991) reported that floral morphology in the *lfy* mutant changes with node number in inflorescences, no experiment has been conducted to explore if such a phenotype results from a gradient of gene expression. In monoecious cucumber, more male flowers are produced at lower nodes and more female flowers at upper nodes (Currence, 1932; Dax-Fuchs et al., 1978). The decreased expression of *CsETR1* and *CsAP3* at upper nodes and the regulatory relationship between CsAP3 and *CsETR1* seems to properly explain this trend, considering the correlation of stamen-specific down-regulation of *CsETR1* and female flower development (Wang et al., 2010). While it is not yet known how the expression of *CsAP3* is regulated, the phenomenon that gene expression changes with node number may not be specific to *CsAP3* and its target *CsETR1*, and its significance may not be restricted to unisexual flower development in cucumber.

Our data also show that the activation activity of CsAP3 on transcription of *CsETR1* is minor although statistically significant. Considering the monoecious property of cucumber and that all three major genes responsible for the unisexual flower development in cucumber encode ACS, the moderate effect of CsAP3 upon *CsETR1* may be crucial for the floral bud at a bipotential stage. The plant would need to sense the internal and external environment sensitively and flexibly and maintain a beneficial balance of male and female ratio of flowers within a single plant. In fact, the connection of a minor decrease in expression of the ethylene receptor with critical developmental events exists not only in cucumber as ethylene-induced stamen-specific DNA damage, but also in the *Aponogeton madagascariensis* (lace plant), where it results in perforations in leaves through programmed cell death (Rantong et al., 2015). These phenomena highlight that in some situations, moderate regulation of gene expression may play important roles in biological processes.

## Author Contributions

J-JS, FL, D-HW, X-FL, XL, NL, H-TG, and CZ, conducted the experiments; J-JS, FL, J-CL, C-XH, S-WH, Z-XZ, Z-HX, and S-NB, designed the experiments; J-JS, and S-NB, wrote the manuscript.

## Conflict of Interest Statement

The authors declare that the research was conducted in the absence of any commercial or financial relationships that could be construed as a potential conflict of interest.

## Footnotes

1 http://www.phytozome.net

2 http://blast.ncbi.nlm.nih.gov

3 http://cucumber.genomics.org.cn

4 http://www.megasoftware.net
