# Supplementary material for: CsAP3: A Cucumber Homolog to Arabidopsis APETALA3 with Novel Characteristics
Source: Front Plant Sci. 2016 Aug 4;7:1181. doi: 10.3389/fpls.2016.01181 (PMC4972961; doi:10.3389/fpls.2016.01181)
Supplement: Supplementary file 1 [file Data_Sheet_1.DOCX]

Supplementary Material

*CsAP3*: a cucumber B-class gene with novel characteristics of decreased expression along stem and activating *CsETR1* promoter with a GV repeat

Jin-Jing Sun^1, 2, 4, 6^, Feng Li^1, 2, 6^, Dong-Hui Wang^1, 2^, Xiao-Feng Liu ^5^, Xia Li^2^, Na Liu^1, 2^, Hai-Tao Gu^1, 2^, Cheng Zou^1, 2^, Jing-Chu Luo^2^, Chao-Xing He^4^, San-Wen Huang^4^, Xiao-Lan Zhang^5^, Zhi-Hong Xu^1, 2^ and Shu-Nong Bai^1, 2, 3,*^

*** Correspondence:** Shu-Nong Bai: shunongb@pku.edu.cn


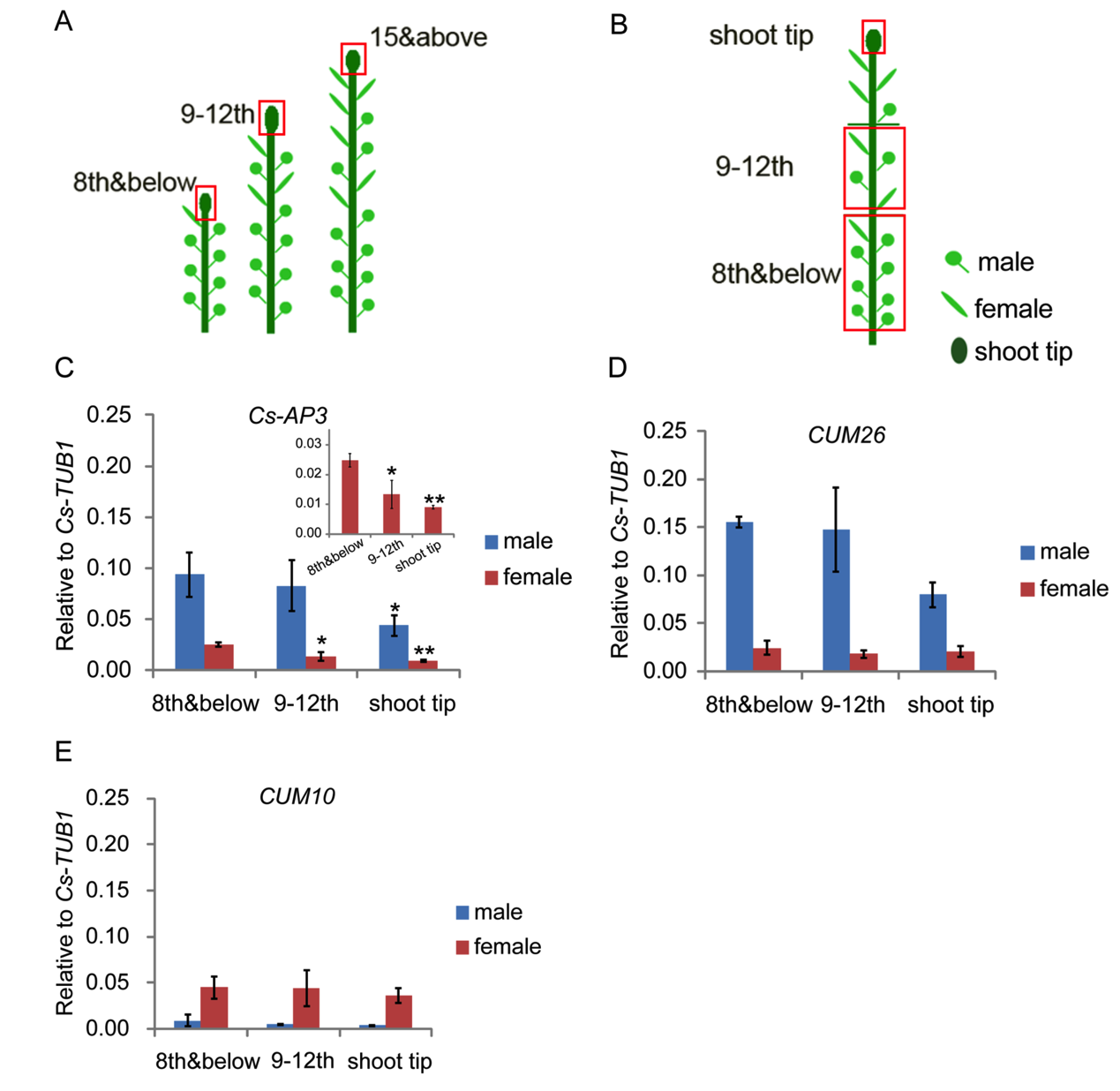


**Supplementary Figure 1.** **Sample collecting methods and *CsAP3* expression level in flowers from different nodes of cucumber.**

**(A)** Floral buds at stage 6-8 were collected from plant grown to designated nodes (boxed). The floral buds at various nodes were therefore collected from different plants.

**(B)** Floral buds at stage 6-8 from designated nodes in plants grown to more than 15 nodes were collected (boxed). So the comparison was between flowers at different nodes but from the same plants.

**(C)** to **(E)** Expression levels of *CsAP3***(C)**, *CUM26* **(D)** and *CUM10* **(E)** in male and female floral buds collected at nodes 8^th^ and below, 9-12^th^, and shoot tip above 15^th^, showing that as expected, B-class genes *CsAP3* and *CUM26* express higher in male than in female flower and C-class gene *CUM10* expresses higher in female than in male flower. Expression of *CsAP3* decreases when nodes increase, not only in male, but more significantly in female flowers. These results were obtained with samples collected method 2 and consistent with the results shown in Fig7, which was obtained with samples collected method 1.

**
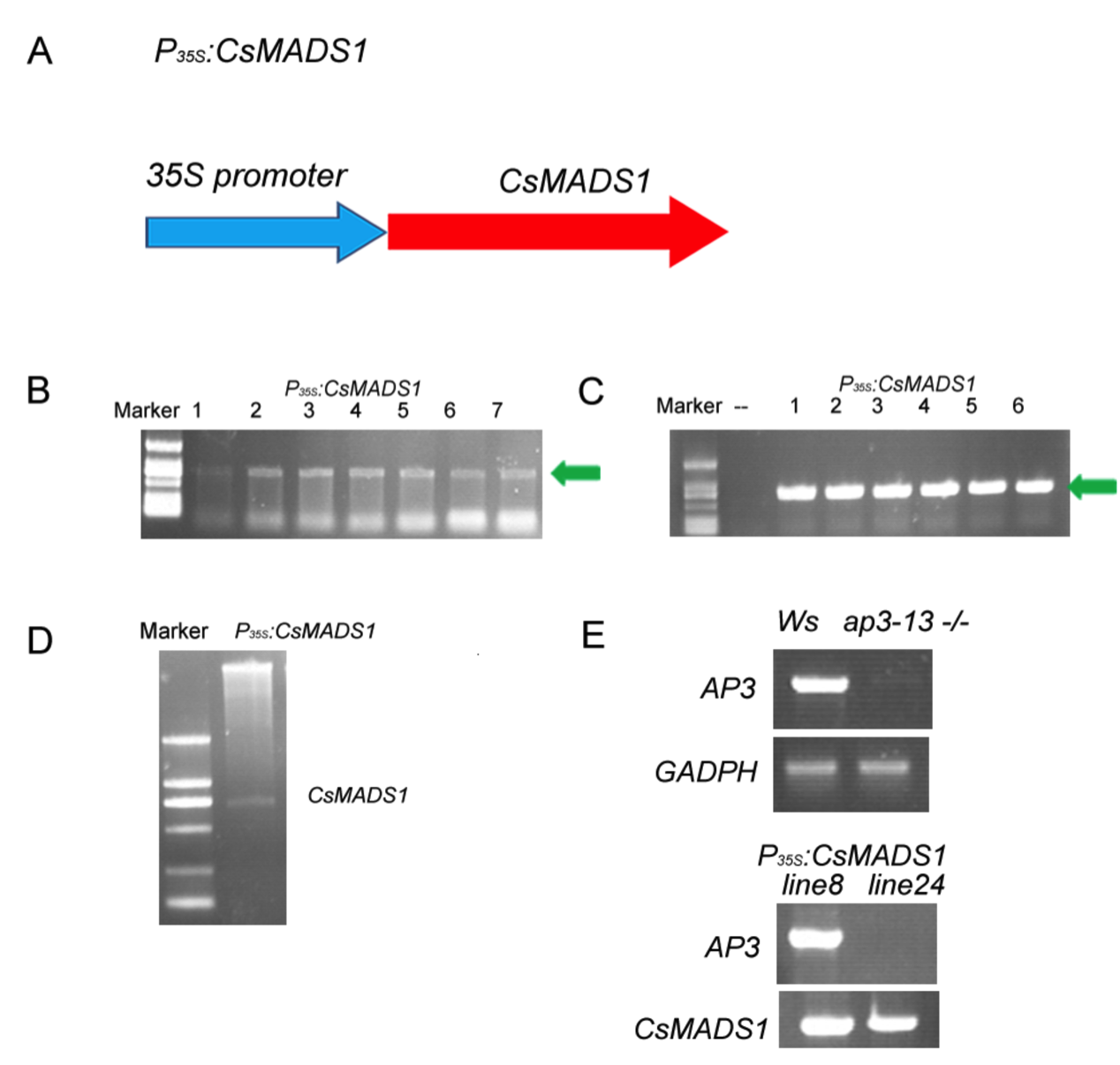
**

**Supplementary Figure 2. Construction and confirmation of the transgenic *P_35S_:CsMADS1*** **plants.**

**(A)** Scheme of the *P_35S_:CsMADS1* constructs.

**(B)** PCR verification of the integrated *P_35S_:CsMADS1* in *E.coli.* Lane1-7: PCR products from individual clones respectively. Green arrow pointed the CsMADS1 bands. M: DL2000 marker.

**(C)** PCR verification of the integrated *P_35S_:CsMADS1* in *A.tumefaciens* - GV3101. Lane 0-6: PCR products from individual clones respectively. Line 0 was negative control. Green arrow pointed the CsMADS1 bands. M: DL2000 marker.

**(D)** Verification of *P_35S_:CsMADS1* plasmid by digested with NcoI and SpeI. M: DL2000 marker.

**(E)** RT-PCR verification of the transgenic plants. Ws and *ap3-13* -/- was the positive and negative control, GADPH was the input control. RT-PCR test were performed in the transgenic lines *P_35S_:CsMADS1*-8 (Ws background) and *P_35S_:CsMADS1*-24 (*ap3-13* -/- background). All the plants were grown in LD condition. Primer sequences used to detect *CsMADS1* were list in Table S3.

**
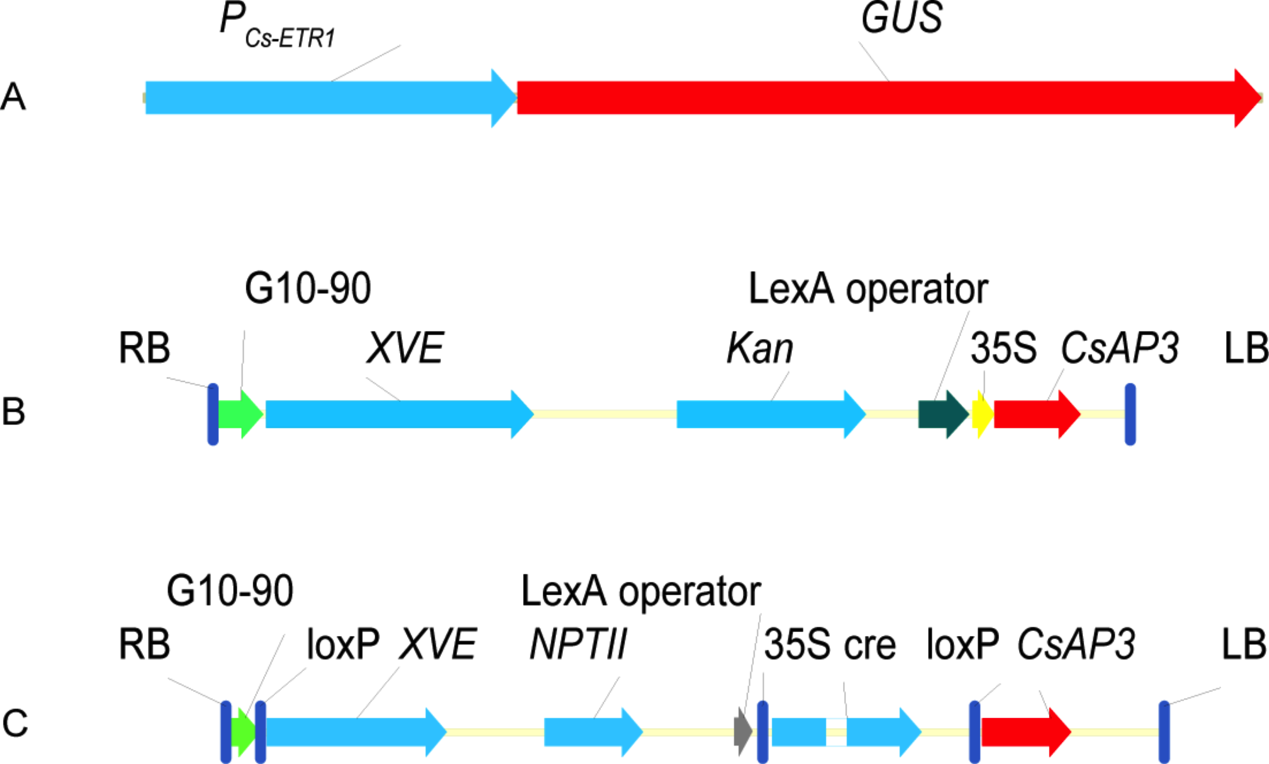
**

**Supplementary Figure 3. Constructs used in the estrogen induced transgenic Arabidopsis system.**

**(A)** Promoter of *CsETR1* was cloned into *pCAMBIA 1305.1*, with the *GUS* as the reporter gene.

**(B)** and **(C)** *CsAP3* was cloned into estrogen induced expression vector *pER10* **(B)** and *PX6* (C). Then the vector with *CsAP3* was transformed into the transgenic line containing *P_CsETR1_:GUS* construct already.

**
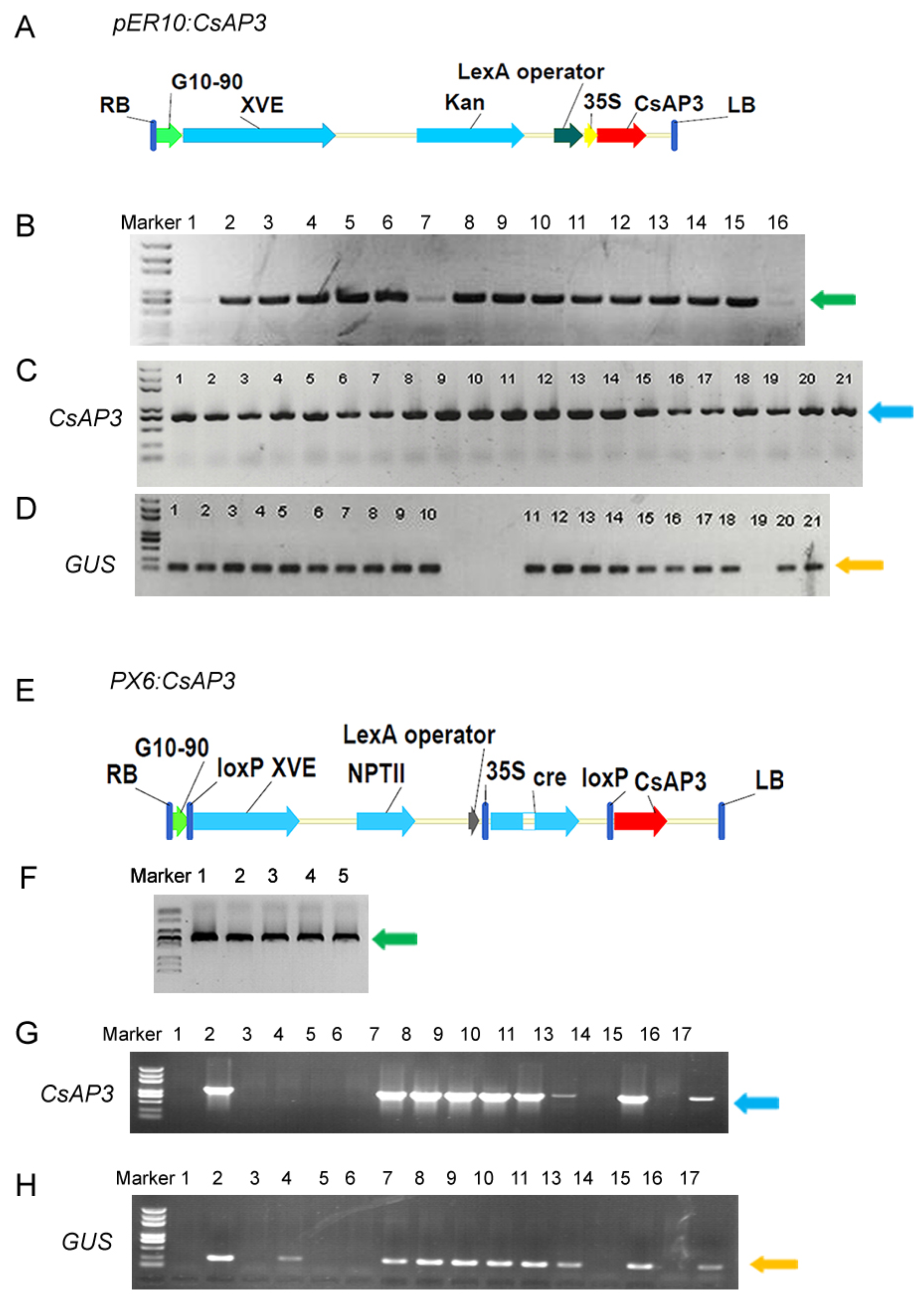
**

**Supplementary Figure 4. Construction and confirmation of estrogen induced transgenic plants.**

**(A)** Scheme of the *pER10:CsAP3* constructs.

**(B)** PCR verification of the integrated *pER10:CsAP3* in *E.coli.* Lane1-15: PCR products from individual clones respectively. Lane 16: negative control. Green arrow pointed *CsAP3* bands. M: DL2000 plus marker.

**(C)** and **(D)** Genomic PCR to verify the transgenic plants. Primers for *CsAP3* (blue arrow pointed) **(C)** and *GUS* (orange arrow pointed) **(D)** were used and genomic DNA from independent *pER10:CsAP3* transgenic lines was used as template.

**(E)** Scheme of the *PX6:CsAP3* constructs.

**(F)** PCR verification of the integrated *PX6:CsAP3* in *E.coli.* Lane1-5: PCR products from individual clones respectively. Green arrow pointed *CsAP3* bands. M: DL2000 marker.

**(G)** and **(H)** Genomic PCR to verify the transgenic plants. Primers for *CsAP3* (blue arrow pointed) **(G)** and *GUS* (orange arrow pointed) **(H)** were used and genomic DNA from independent *PX6:CsAP3* transgenic lines was used as template.


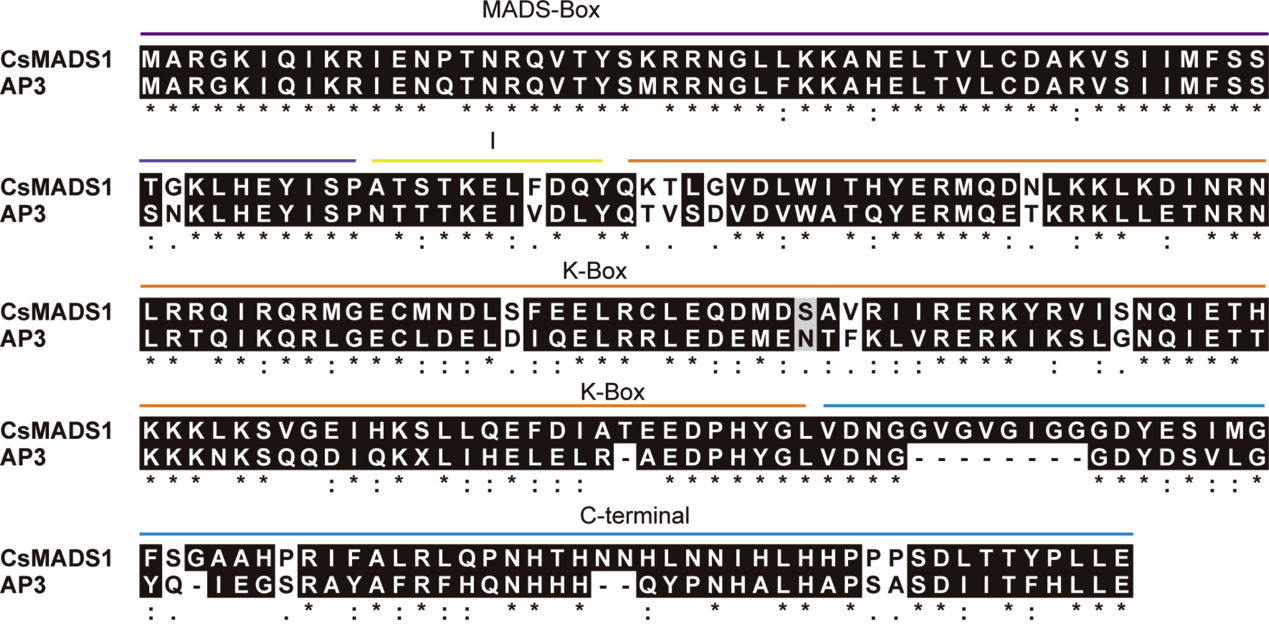


**Supplementary Figure 5. Alignment results of CsMADS1 and AP3 protein sequences.**

Amino acid residues displaying >50% identity or similarity between the two homologs are shaded black or gray, respectively. Conserved MADS-Box, I domain and K-Box were marked with purple, yellow and orange lines. C-terminal was lined with light blue.


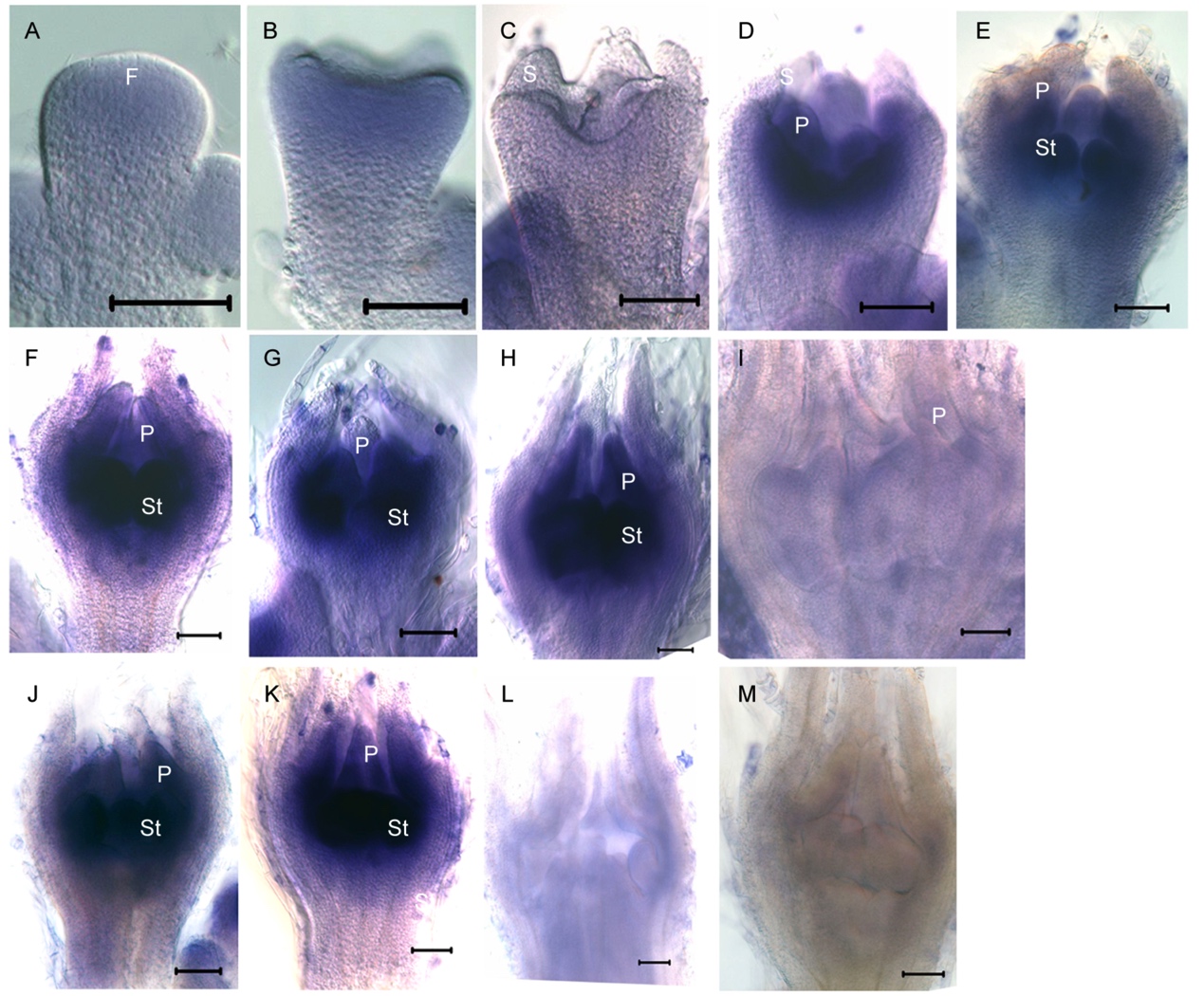


**Supplementary Figure 6. Detection of *CsAP3* with whole-mount *in situ* hybridization.**

*CsAP3* RNA was detected in floral buds at stage 4 and 5 **(D-E)** at the regions where petals and stamens were emerging (**A-C,** stage 1-3 respectively). In male flowers, *CsAP3* expression was detected in petals and stamens at stage 6, 7, 8 (**F-H** respectively) and decreased at stage 9 **(I)**. In female flowers the *CsAP3* expression was detected in petals and stamens at stage 6 and 8 **(J-K)**, but disappeared from stamens after stage 8 **(L)**. **(M)**: negative control. F: floral meristem; S: sepal; P: petal; St: stamen. Bars =100 μm.

**
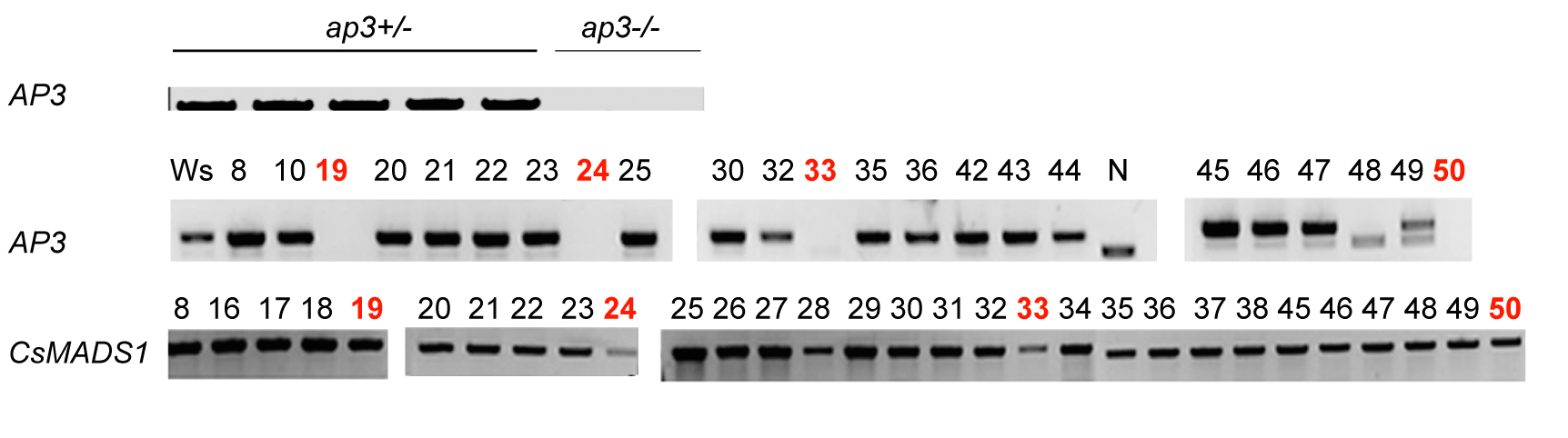
**

**Supplementary Figure 7. Identification of homozygous *ap3-/-* in the transgenic Arabidopsis lines.**

The upper panel showed that primers used in genotyping are effective. The middle panel shows the lines, such as lines 19, 24, 33 and 50, that were homozygous for *ap3* (highlighted with red). The lower panel shows the homozygous *ap3* that were successfully transformed with *CsMADS1* (see the bands in line 19, 24, 33 and 50). Ws: Wassilewskija， N: negative control for PCR without adding the templates.


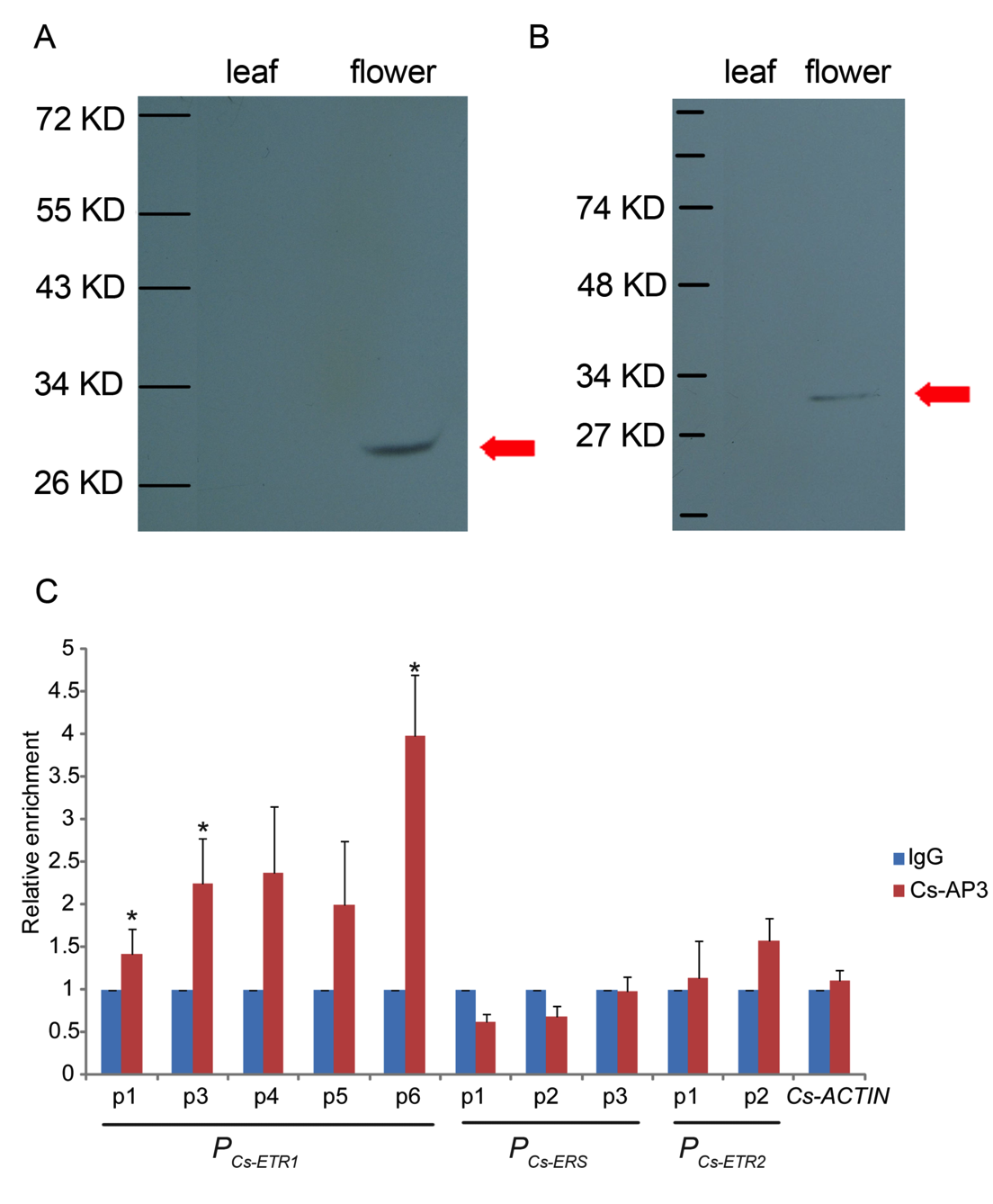


**Supplementary Figure 8. Immunoblot to test the specificity of AP3 antibody.**

**(A)** AP3 antibody (Santa Cruz #sc‐12639) can recognize AP3 protein from Arabidopsis flower. Protein from Col leaf was used as negative control.

**(B)** AP3 antibody (Santa Cruz #sc‐12639) can recognize CsAP3 protein from cucumber flower. Protein from cucumber leaf was used as negative control. CsAP3 and AP3 are indicated with the red arrowheads.

**(C)** ChIP-qPCR showing that CsAP3 significantly enriched fragments of *CsETR1* promoter (p1 to p4) that contain a CArG box but not p5. CsAP3 also significantly enriched fragment p6 although it contains no predicted CArG box. CsAP3 did not significantly enrich fragments containing CArG boxes of the *CsERS* promoter or *CsETR2.* The ChIP results are presented as relative enrichment to *CsTUBLIN*. Error bars indicate SD (n = 3). * indicate p<0.05 in Student’s *t*-test.


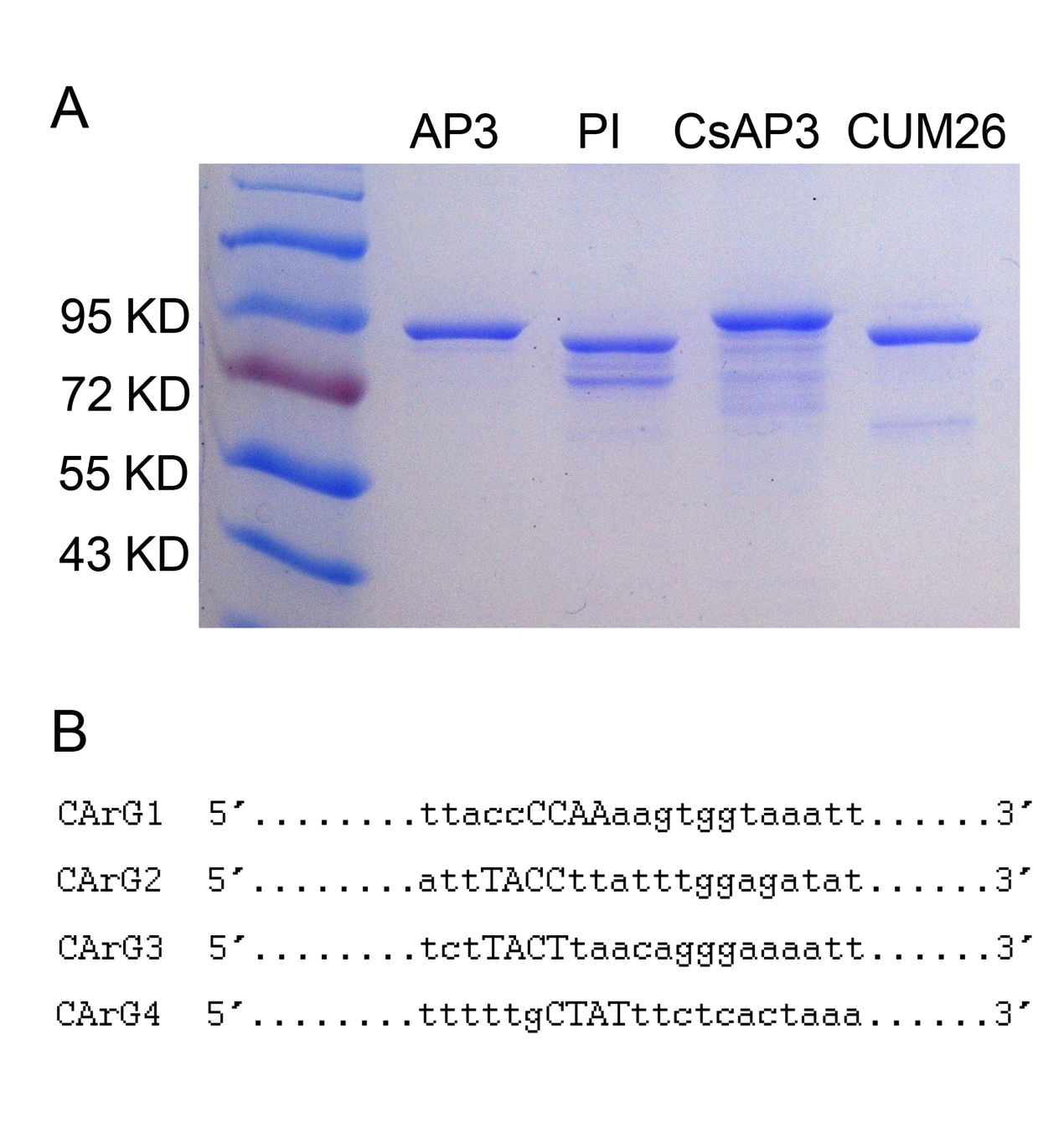


**Supplementary Figure 9. SDS-PAGE of recombinant proteins of CsAP3, CUM26, AP3, PI and core sequence of probes used in EMSA assay.**

SDS-PAGE of purified recombinant proteins. Lane 1: AP3 protein. Lane 2: PI protein. Lane 3: CsAP3 protein. Lane4: CUM26 protein. The proteins were visualized by staining with coomassie brilliant blue R250. The concentration was adjusted to almost the same to be used in EMSA assay.


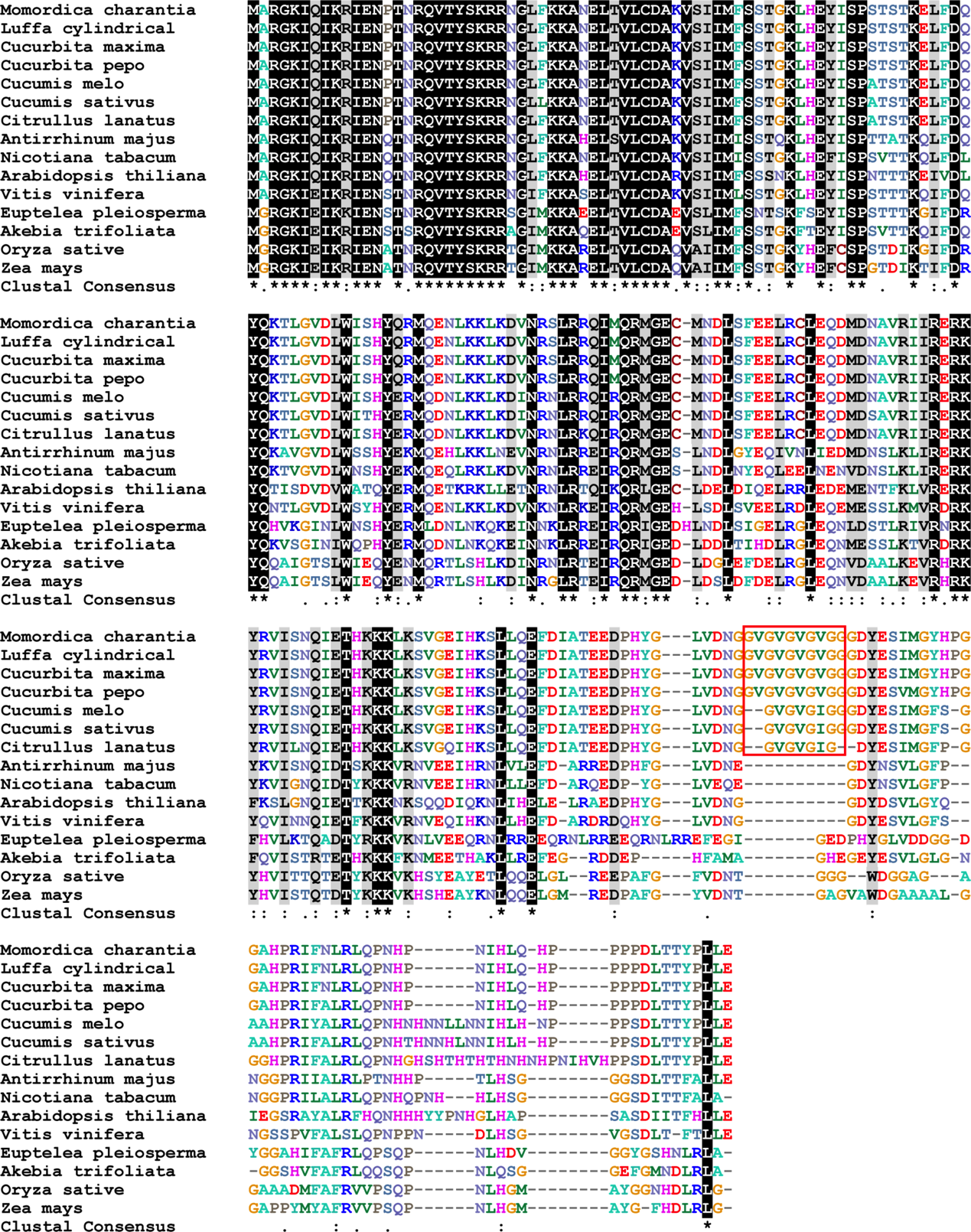


**Supplementary Figure 10. Alignment Results of AP3 homologs in core eudicots, basal eudicots and monocots.**

Amino acid residues displaying >100% identity or similarity among the homologs are shaded black or gray, respectively. Conserved MADS-Box, I domain and K-Box were marked with purple, brown and orange lines, respectively. C-terminal was lined with light blue. Red rectangle shows the additional GV repeated sequence in bitter guard (*Monordica Charantia*), Sponge gouard (*Luffa cylindrical*), *cucurbita maxima*, *cucurbita pepo*, cucumber (*Cucumis sativus*), melon (*cucumis melo*) and watermelon (*Citrullus laratus*).

**Table S1. Genes used in the phylogenetic analysis.**

| Class | order | SpeciesS | Name | Accesion number |
| --- | --- | --- | --- | --- |
| monocots | Graminales | *Oryza sativa* | Os_MADS16 | NP_001058560 |
|  |  | *Zea mays* | Zm_MADS16 | ACG37775 |
| eudicots | Scrophulariaceae | *Antirrhinum majus* | Am_AP3 | CAA36268 |
|  | Vitales | *Vitis vinifera* | Vv_AP3 | ABN71371 |
|  | Malpighiales | *Populus tomentosa* | Pt_AP3 | AAO49713 |
|  | Brassicales | *Arabidopsis thaliana* | AP3 | NP_191002 |
|  |  |  | AGL71 | NP_974924 |
|  |  |  | AGL8 | NP_568929 |
|  |  |  | AGL6 | NP_182089 |
|  |  |  | AGL20 | NP_182090 |
|  |  |  | PI | NP_197524 |
|  |  |  | AG | NP_567569 |
|  |  |  | AP1 | NP_177074 |
|  |  |  | STK | NP_192734 |
|  |  |  | SEP4 | NP_178466 |
|  |  |  | SHP1 | NP_001078311 |
|  |  |  | SHP2 | NP_850377 |
|  |  |  | SEP2 | NP_186880 |
|  |  |  | SEP1 | NP_568322 |
|  |  |  | AGL13 | NP_191671 |
|  |  |  | AGL15 | NP_196883 |
|  |  |  | AGL44 | NP_179033 |
|  |  |  | CAL | NP_564243 |
|  |  |  | AGL14 | NP_192925 |
|  |  |  | AGL21 | NP_195507 |
|  |  |  | AGL16 | NP_191282 |
|  |  |  | AGL19 | NP_194026 |
|  |  |  | AGL72 | NP_199999 |
|  |  |  | SEP3 | NP_564214 |
|  |  |  | AGL17 | NP_179848 |
|  |  |  | TT16 | NP_974823 |
|  |  |  | SVP | NP_179840 |
|  |  |  | AGL24 | NP_194185 |
|  |  |  | AGL79 | NP_189645 |
|  |  |  | AGL42 | NP_568952 |
|  |  |  | AGL18 | NP_191298 |
|  |  |  | FLC | AAX51271 |
|  | Cucurbitaceae | *Momordica charantia* | Mc_ABE0388 | ABE03878 |
|  |  |  | Mc_ABC2554 | ABC25564 |
|  |  |  | Mc_AAO2014 | AAO20104 |
|  |  | *Cucumis sativus* | Csa3M865440 | Csa3M865440 |
|  |  |  | Csa6M07671 | Csa6M07671 |
|  |  |  | Csa6M367090 | Csa6M367090 |
|  |  |  | Csa1M051580 | Csa1M051580 |
|  |  |  | Csa1M039910 | Csa1M039910 |
|  |  |  | Csa6M095270 | Csa6M095270 |
|  |  |  | Csa6M076720 | Csa6M076720 |
|  |  |  | Csa1M467100 | Csa1M467100 |
|  |  |  | Csa1M039900 | Csa1M039900 |
|  |  |  | Csa4M126990 | Csa4M126990 |
|  |  |  | Csa5M156170 | Csa5M156170 |
|  |  |  | Csa6M367080 | Csa6M367080 |
|  |  |  | Csa4M126480 | Csa4M126480 |
|  |  |  | Csa3M686200 | Csa3M686200 |
|  |  |  | Csa4M410880 | Csa4M410880 |
|  |  |  | Csa5M172800 | Csa5M172800 |
|  |  |  | Csa6M499000 | Csa6M499000 |
|  |  |  | Csa5M172800 | Csa5M172800 |
|  |  |  | Csa3M893290 | Csa3M893290. |
|  |  |  | Csa2M370550 | Csa003859 |
|  |  |  | Cs_AAD0220 | AAD02250 |
|  |  |  | CUM10 | AAC08529 |
|  |  |  | Cs_CAC37399 | CAC37399 |
|  |  |  | CUM1 | AAC08528 |
|  |  |  | SLM3 | CAA56657 |
|  | Caryophyllaceae | *Silene latifolia* | SLM2 | CAA56656 |
|  |  |  | Sl_BAD10944 | BAD10944 |
|  |  |  | SLM1 | CAA56655 |
|  |  |  | SLM5 | CAA56659 |
|  |  |  | SLM4 | CAA56658 |
|  |  |  | Sl_BAD10945 | BAD10945 |
|  |  |  | ScAP3A | BAC77705 |
|  |  | *Silene conica* | Ra_CAA6144 | CAA61484 |
|  | Polygonaceae | *Rumex acetosa* | Ra_CAA6141 | CAA61481 |
|  |  |  | Ra_CAA6140  CAA61480 | |

**Table S2. Gene specific primers used in this study.**

| Gene name | | | Primer name | | | sequence (5' to 3') |
| --- | --- | --- | --- | --- | --- | --- |
| ChIP-qPCR assays | | | | | | |
| CsETR1 | | | CsETR1p1_F | | TCTTTTAATCATAGGACCAAACTAATTAGG | |
|  |  |  | CsETR1p1_R | | GTAAGGGTTAGGCCGTGATCGTA | |
|  |  |  | CsETR1p2_F | | AAATGTTTACGATCACGGCCTA | |
|  |  |  | CsETR1p2_R | | TTTAGTTAGTATACACTTCTAAAATCGTGATAAT | |
|  |  |  | CsETR1p3_F | | ATTAAATTATCACGATTTTAGAAGTGTATACTAAC | |
|  |  |  | CsETR1p3_R | | TTTTCCCTGTTAAGTAAGATGGTGG | |
|  |  |  | CsETR1p4_F | | CCACCATCTTACTTAACAGGGAAAA | |
|  |  |  | CsETR1p4_R | | TCTTCACTCCGCCCTCTATTCTTTA | |
|  |  |  | CsETR1p5_F | | TTAAAGAATAGAGGGCGGAGTGAAG | |
|  |  |  | CsETR1p5_R | | ACGCTAACCCCTCTCTCTCCACA | |
|  |  |  | CsETR1p6_F | | AAATGTGGAGAGAGAGGGGTTAGCG | |
|  |  |  | CsETR1p6_R | | GGTTTTGGGAGTTCTGAGACAAGCA | |
| CsETR2 | | | CsETR2p5_F | | CCCAACTTCACATACAACACTCTACC | |
|  |  |  | CsETR2p5_R | | GAAAAGGAGAAAGCAAGAAAAACA | |
|  |  |  | pCsETR2_365F | | TGGATGGTTTGTATATATTTGCAGAGTCTA | |
|  |  |  | pCsETR2_499R | | TCTCATTTATTTAGTTTATTAATATTGCATG | |
| CsERS | | | pCsERS _309F | | AAAGTAATCATTTCTTTTATCACAATCTCAAC | |
|  |  |  | pCsERS 420R | | TTTATAAGTAGTGAAAGGTGATTAATTTGGAA | |
|  |  |  | pCsERS 548F | | TTTTATAACTAATAAATTCTTAAAGTTTCAATTT | |
|  |  |  | pCsERS 659R | | AACAACCGTGAACAATTCATATGTTG | |
|  |  |  | CsERSp1F | | ACATATGAATTGTTCACGGTTGTTCTAG | |
|  |  |  | CsERSp1R | | CACGTGTGAAAATAACCTATCAAATAGTT | |
| ETR1 | | | ETR1p1F | | ATGGGATTAGAAGTGAAAATTGTAACC | |
|  |  |  | ETR1p1R | | AAGAATAAAAATCGCAATACCAAAATA | |
|  |  |  | ETR1p2F | | TAGTATTGGCCCCACTCTTTCCC | |
|  |  |  | ETR1p2R | | ATTTTATGGATTTCCAAATTAAATCTAATT | |
|  |  |  | ETR1p3F | | TAATGTTGAGACCACAAAATTCTATTAAT | |
|  |  |  | ETR1p3R | | TCTTGTTGTTTTCTAGACATGATAATT | |
|  |  |  | ETR1p4F | | ATTATCATGTCTAGAAAACAACAAGAAAAG | |
|  |  |  | ETR1p4R | | TTGAAAATCTTATATAAACATAGGGTCCCA | |
|  |  |  | ETR1p5F | | TTGTATCACTTTTTACAGTGGTTCCAACTC | |
|  |  |  | ETR1p5R | | GCTCTAATCAGCCTATCTCGAACTG | |
|  |  |  | ETR1p6F | | AAATGAGATTCAGTTCGAGATAGGCT | |
|  |  |  | ETR1p6R | | AACCAGTCAAATCAAAATCCAAACA | |
|  |  |  | ETR1p7F | | TGTTTGGATTTTGATTTGACTGGTT | |
|  |  |  | ETR1p7R | | TTCAGTACCTCAAAACTAAATCAGAGTTAA | |
|  |  |  | ETR1p8F | | GGTTAACTCTGATTTAGTTTTGAGGTACTG | |
|  |  |  | ETR1p8R | | CAAACAAATATGTTCATGTGAATAAAAGC | |
|  |  |  | ETR1p9F | | TGTTTGATTCAACTTGTTTTACTTCTCTT | |
|  |  |  | ETR1p9R | | CACTAACTTTCACCTTACATTGATTCGT | |
|  |  |  | ETR1p10F | | ACGAATCAATGTAAGGTGAAAGTTAGTG | |
|  |  |  | ETR1p10R | | ATAGCTGGTGGGTGCATGAATT | |
|  |  |  | ETR1p11F | | CAAATTCATGCACCCACCAGC | |
|  |  |  | ETR1p11R | | CGACGGAGAAGGAGAAAAGGG | |
|  |  |  | ETR1p12F | | CAACGCTCCCCTTTTCTCCTTC | |
|  |  |  | ETR1p12R | | TGAGATCGACGACAACAACAGAAGA | |
|  |  |  | ETR1p13F | | CTTCTGTTGTTGTCGTCGATCTC | |
|  |  |  | ETR1p13R | | AAATAGGTTGCATATTTCAGTCTCG | |
|  |  |  | ETR1p14F | | ACCTATTTTTTCGTAATCGTTGTGA | |
|  |  |  | ETR1p14R | | ATAGTTCCTCTCTCACACATACACACAC | |
| AP1 | | | pAP1_F | | GGAAATCTCCGCCGTCAAT | |
|  |  |  | pAP1_R | | TGGTCCTTCCCCAAGTGTCA | |
| AP3 | | | pAP3_F | | TTACCTTTCATGGATTAGGCAATACTT | |
|  |  |  | pAP3_R | | GGGTCCACTTGAGTTACTAAAAATGG | |
| qRT-PCR analyses | | | | | | |
| CsMADS1 | | | CsMADS1_rtF | | GTTCTCAGGTGCTGCCCATC | |
|  |  |  | CsMADS1_rtR | | TCTGAAGGAGGAGGGTGATG | |
| CUM26 | | | CUM26_rtF | | TGGCCTCACCGGTGTTCGTGA | |
|  |  |  | CUM26_rtR | | AGGCGCTTGTTCTCTTCTTCCATCA | |
| CUM10 | | | CUM10_rtF | | CTCCAGCCGTGGCCGTCTCT | |
|  |  |  | CUM10_rtR | | CGCAGTTTAGCCGATTCTTGCTGA | |
| CsACTIN2 | | | CsACTIN2_F | | GTTACGCCCTCCCTCATGCCATTC | |
|  |  |  | CsACTIN2_R | | TCCCGTTCGGCAGTGGTGGT | |
| CsTUBLIN1 | | | CsTUB1_F | | ACCGTTGGAAAGGAAATTGTTG | |
|  |  |  | CsTUB1_R | | GGAGCCGAGACCAGAACC | |
| GUS | | | GUS_rtF | | AAGGAAACAAGAAGGGCGTG | |
|  |  |  | GUS_rtR | | CGTGATGGTGATGGTGATGG | |
| UBIQUITIN | | | UBQ_rtF | | GTACTTTGGCGGATTACAACATC | |
|  |  |  | UBQ_rtR | | GAATACCTCCTTGTCCTGGATCT | |
| *In situ* expression analyses | | | | | | |
| CsAP3 | CsAP3_sense_T7 | | | | AATTAATACGACTCACTATAGGG AGAACTCCGATGTCTTGAGCAAGAT | |
|  | CsAP3_antisense | | | | TATTAAGATGGTTGTTGTGGGTGTG | |
|  | CsAP3_sense | | | | AGAACTCCGATGTCTTGAGCAAGAT | |
|  | CsAP3_antisenseT7 | | | | AATTAATACGACTCACTATAGGG TATTAAGATGGTTGTTGTGGGTGT | |
| transgenic plant construction | | | | | | |
| CsAP3 | | P35S:CsAP3_F | | | cccccatggCTCGTGGGAAGATCCA | |
|  |  | P35S:CsAP3_R | | | cccactagtCTCAAGGAGTGGATAGGTAG | |
| Yeast one hybrid | | | | | | |
| CsETR1 promoter | | Y1F | | CGCGGAATTCTCTTTTAATCATAGGACCAAACTA | | |
|  |  | Y1R | | CGCGACGCGTGGTGCATTTCGTTAAGTTAACTAG | | |
|  |  | Y2F | | CGCGCGGAATTCGCACCATTGATTAAAATGTT | | |
|  |  | Y2R | | CGCGCG ACGCGTATAGAATCTAGTGAGAAGTTACAA | | |
|  |  | Y3F | | CGCGGAATTCAGTTATTAATTACAAATTGGGAGA | | |
|  |  | Y3R | | CGCGACGCGTGAGGATAGGAGTGATTTAGTTAGT | | |
|  |  | Y4F | | CGGAATTCAAATCACTCCTATCCTCTCACA | | |
|  |  | Y4R | | CGACGCGTCTGTTAAGTAAGATGGTGGGAT | | |
|  |  | Y5F | | CGCGGAATTCGGGAAAATTTTTGTTTACTT | | |
|  |  | Y5R | | CGCGACGCGTTCACCTAACTTCCCTCTTTT | | |
|  |  | Y6F | | CGCGGAATTCGGTTATCTGGAAAAGAGGGA | | |
|  |  | Y6R | | CGCGACGCGTACTCCGCCCTCTATTCTTTA | | |
|  |  | Y7F | | CGCGGAATTCTAATTAAAGAATAGAGGGCG | | |
|  |  | Y7R | | CGCGACGCGTCAGATTCATGCACCATCTTA | | |
|  |  | Y8F | | CGGAATTCTAAGATGGTGCATGAATCTG | | |
|  |  | Y8R | | CGACGCGT CCCTCTCTCTCCACATTTTT | | |
|  |  | Y9F | | CGGAATTCAAAATGTGGAGAGAGAGGGG | | |
|  |  | Y9R | | CGACGCGTAGGAAATTGGGAAGGAAAAC | | |
|  |  | Y10F | | CGGAATTCACTTTCCGTTTGCTTGTCTC | | |
|  |  | Y10R | | CGACGCGTTCTAAATGCAATGTTTGTGG | | |

**Table S3**

**Prediction of transcription factors binding sites of promoter of *CsETR1.***

| Family | Matrix | Opt. threshold | Start pos. | End pos. | Strand | Matrix sim. | Core sim. | Sequence |
| --- | --- | --- | --- | --- | --- | --- | --- | --- |
| O$MTEN | O$DMTE.01 | 0.77 | 957 | 977 | - | 0.772 | 0.938 | acaagcaAACGgaaagtgagg |
| O$TF2D | O$INR_DPE.01 | 0.69 | 133 | 171 | - | 0.731 | 0.848 | ttaatttaccacttttggggtaagggttagGCCGtgatc |
| O$TF3C | O$TFIIIC.01 | 0.87 | 387 | 397 | - | 0.881 | 1 | GGTTggaaaca |
| O$VTBP | O$ATATA.01 | 0.78 | 77 | 93 | - | 0.845 | 0.75 | tagtattTAATctcgta |
| O$VTBP | O$VTATA.02 | 0.89 | 430 | 446 | - | 0.904 | 1 | atgcaTAAAatttctcc |
| O$VTBP | O$ATATA.01 | 0.78 | 606 | 622 | + | 0.825 | 1 | ccctagtTAAGggggaa |
| O$VTBP | O$VTATA.02 | 0.89 | 694 | 710 | + | 0.89 | 1 | gtacaTAAAatagggtc |
| O$VTBP | O$VTATA.01 | 0.9 | 734 | 750 | - | 0.924 | 1 | tgctaTAAAgttgcttc |
| O$VTBP | O$ATATA.01 | 0.78 | 745 | 761 | - | 0.797 | 0.75 | ctgtaatTAAAtgctat |
| P$AHBP | P$WUS.01 | 0.94 | 1 | 11 | + | 0.963 | 1 | tctttTAATca |
| P$AHBP | P$HAHB4.01 | 0.87 | 5 | 15 | - | 0.925 | 1 | cctatgATTAa |
| P$AHBP | P$BLR.01 | 0.9 | 55 | 65 | + | 0.936 | 0.826 | gaaGTTAttat |
| P$AHBP | P$WUS.01 | 0.94 | 81 | 91 | - | 0.963 | 1 | gtattTAATct |
| P$AHBP | P$WUS.01 | 0.94 | 117 | 127 | - | 0.963 | 1 | catttTAATca |
| P$AHBP | P$BLR.01 | 0.9 | 164 | 174 | + | 1 | 1 | taaATTAattt |
| P$AHBP | P$BLR.01 | 0.9 | 165 | 175 | - | 1 | 1 | aaaATTAattt |
| P$AHBP | P$WUS.01 | 0.94 | 299 | 309 | - | 0.963 | 1 | taattTAATct |
| P$AHBP | P$BLR.01 | 0.9 | 488 | 498 | - | 0.92 | 0.826 | gaaTTTAgtga |
| P$AHBP | P$HAHB4.01 | 0.87 | 499 | 509 | + | 0.909 | 1 | tacattATTAt |
| P$AHBP | P$BLR.01 | 0.9 | 636 | 646 | - | 0.92 | 0.826 | gaaTTTAgtga |
| P$AHBP | P$ATHB5.01 | 0.89 | 645 | 655 | + | 0.919 | 1 | tccATTAttta |
| P$AHBP | P$WUS.01 | 0.94 | 795 | 805 | - | 0.963 | 1 | catttTAATct |
| P$ASRC | P$AS1_AS2_II.01 | 0.86 | 926 | 934 | + | 0.906 | 1 | acgTTGAct |
| P$CCAF | P$EE.01 | 0.84 | 176 | 190 | + | 0.863 | 1 | gagagaAATAtttac |
| P$CCAF | P$EE.01 | 0.84 | 179 | 193 | - | 0.864 | 1 | ttagtaAATAtttct |
| P$CCAF | P$CCA1.01 | 0.85 | 218 | 232 | - | 0.866 | 1 | aactatagAATCtag |
| P$CCAF | P$EE.01 | 0.84 | 430 | 444 | - | 0.863 | 0.75 | gcataaAATTtctcc |
| P$CCAF | P$CCA1.01 | 0.85 | 468 | 482 | - | 0.855 | 0.757 | taaacaaaAATTttc |
| P$CCAF | P$EE.01 | 0.84 | 812 | 826 | - | 0.851 | 1 | tctcatAATAtttta |
| P$CCAF | P$CCA1.01 | 0.85 | 840 | 854 | + | 0.854 | 0.767 | taaaaaaaAATGtgg |
| P$CCAF | P$EE.01 | 0.84 | 934 | 948 | - | 0.869 | 0.75 | aaggaaAACAtctaa |
| P$CGCG | P$OSCBT.01 | 0.78 | 994 | 1010 | - | 0.808 | 0.817 | tttCGGGtgtggtgggg |
| P$CNAC | P$CBNAC.02 | 0.85 | 968 | 988 | + | 0.891 | 1 | gtttGCTTgtctcagaactcc |
| P$DOFF | P$DOF1.01 | 0.98 | 282 | 298 | + | 0.98 | 1 | agtttattAAAGgttta |
| P$DOFF | P$PBOX.01 | 0.75 | 367 | 383 | - | 0.798 | 1 | tccattggAAAGagtat |
| P$DOFF | P$PBOX.01 | 0.75 | 375 | 391 | + | 0.766 | 1 | tccaatggAAAGtgttt |
| P$DOFF | P$DOF1.01 | 0.98 | 654 | 670 | + | 0.994 | 1 | tattaattAAAGaatag |
| P$DREB | P$CRT_DRE.01 | 0.89 | 915 | 935 | + | 0.924 | 1 | gagatgaaCCGAcgttgactt |
| P$DREB | P$RAP2.1.01 | 0.77 | 1043 | 1063 | + | 0.81 | 0.75 | aaaaaacaCCCAcaaacattg |
| P$EINL | P$TEIL.01 | 0.92 | 691 | 699 | - | 0.935 | 1 | aTGTActtt |
| P$EINL | P$TEIL.01 | 0.92 | 778 | 786 | + | 0.972 | 0.964 | aTGAAtctg |
| P$GAGA | P$BPC.01 | 1 | 851 | 875 | + | 1 | 1 | gtggagAGAGaggggttagcgttaa |
| P$GAGA | P$GAGABP.01 | 0.75 | 898 | 922 | + | 0.801 | 1 | ggaaatAGAGtgcgagagagatgaa |
| P$GAGA | P$GAGABP.01 | 0.75 | 900 | 924 | + | 0.784 | 0.75 | aaatagAGTGcgagagagatgaacc |
| P$GAGA | P$BPC.01 | 1 | 908 | 932 | + | 1 | 1 | tgcgagAGAGatgaaccgacgttga |
| P$GAGA | P$BPC.01 | 1 | 1013 | 1037 | + | 1 | 1 | agttagAGAGagaaacatgaaattc |
| P$GAGA | P$BPC.01 | 1 | 1015 | 1039 | + | 1 | 1 | ttagagAGAGaaacatgaaattcaa |
| P$GAPB | P$GAP.01 | 0.88 | 516 | 530 | + | 0.88 | 1 | gaagATGAaaaggtt |
| P$GTBX | P$GT1.01 | 0.85 | 52 | 68 | + | 0.894 | 1 | tttgaaGTTAttatttt |
| P$GTBX | P$SBF1.01 | 0.87 | 61 | 77 | - | 0.873 | 1 | acatttaTTAAaataat |
| P$GTBX | P$SBF1.01 | 0.87 | 77 | 93 | + | 0.876 | 1 | tacgagaTTAAatacta |
| P$GTBX | P$SBF1.01 | 0.87 | 78 | 94 | - | 0.876 | 1 | ctagtatTTAAtctcgt |
| P$GTBX | P$SBF1.01 | 0.87 | 113 | 129 | + | 0.883 | 1 | ccattgaTTAAaatgtt |
| P$GTBX | P$GT1.01 | 0.85 | 157 | 173 | + | 0.859 | 0.969 | aaagtgGTAAattaatt |
| P$GTBX | P$GT1.01 | 0.85 | 180 | 196 | - | 0.893 | 0.969 | gatttaGTAAatatttc |
| P$GTBX | P$GT3A.01 | 0.83 | 201 | 217 | - | 0.878 | 1 | tgagaaGTTAcaaaatt |
| P$GTBX | P$SBF1.01 | 0.87 | 227 | 243 | + | 0.913 | 1 | atagttaTTAAttacaa |
| P$GTBX | P$SBF1.01 | 0.87 | 228 | 244 | - | 0.933 | 1 | tttgtaaTTAAtaacta |
| P$GTBX | P$SBF1.01 | 0.87 | 295 | 311 | + | 0.892 | 1 | tttaagaTTAAattatc |
| P$GTBX | P$GT1.01 | 0.85 | 325 | 341 | - | 0.866 | 1 | gatttaGTTAgtataca |
| P$GTBX | P$ASIL1.01 | 0.93 | 333 | 349 | - | 0.933 | 1 | ataggaGTGAtttagtt |
| P$GTBX | P$GT1.01 | 0.85 | 416 | 432 | + | 0.973 | 0.969 | aataagGTAAatttgga |
| P$GTBX | P$GT1.01 | 0.85 | 576 | 592 | + | 0.872 | 0.844 | agttagGTGAaaatttg |
| P$GTBX | P$S1F.01 | 0.79 | 637 | 653 | - | 0.809 | 1 | aataATGGaatttagtg |
| P$GTBX | P$SBF1.01 | 0.87 | 746 | 762 | - | 0.914 | 1 | actgtaaTTAAatgcta |
| P$HEAT | P$HSFA1A.01 | 0.75 | 426 | 442 | - | 0.784 | 1 | ataaaaTTTCtccaaat |
| P$HEAT | P$HSE.01 | 0.81 | 727 | 743 | - | 0.864 | 1 | aagttgcttcaAGAAtc |
| P$HMGF | P$HMG_IY.01 | 0.89 | 646 | 660 | + | 0.897 | 1 | ccatTATTtattaat |
| P$HMGF | P$HMG_IY.01 | 0.89 | 808 | 822 | - | 0.935 | 1 | ataaTATTttatttt |
| P$IBOX | P$GATA.01 | 0.93 | 879 | 895 | + | 0.949 | 1 | aggtgGATAaaatgggg |
| P$IDRS | P$IDRS.01 | 0.69 | 985 | 1011 | + | 0.701 | 1 | ctcccaaaaccccaCCACacccgaaaa |
| P$L1BX | P$ATML1.01 | 0.82 | 65 | 81 | + | 0.979 | 1 | ttttaaTAAAtgtacga |
| P$L1BX | P$HDG9.01 | 0.77 | 79 | 95 | + | 0.861 | 1 | cgagatTAAAtactagt |
| P$L1BX | P$HDG9.01 | 0.77 | 297 | 313 | + | 0.831 | 1 | taagatTAAAttatcac |
| P$L1BX | P$ATML1.02 | 0.76 | 486 | 502 | + | 0.832 | 0.808 | tctCACTaaattctaca |
| P$L1BX | P$PDF2.01 | 0.85 | 634 | 650 | + | 0.875 | 1 | tctcacTAAAttccatt |
| P$L1BX | P$PDF2.01 | 0.85 | 744 | 760 | - | 0.902 | 1 | tgtaatTAAAtgctata |
| P$L1BX | P$ATML1.02 | 0.76 | 746 | 762 | + | 0.827 | 1 | tagCATTtaattacagt |
| P$LEGB | P$RY.01 | 0.87 | 762 | 788 | - | 0.887 | 1 | ttcagattCATGcaccatcttaatata |
| P$LFYB | P$LFY.01 | 0.93 | 111 | 123 | + | 0.947 | 0.914 | cACCAttgattaa |
| P$LREM | P$RAP22.01 | 0.85 | 356 | 366 | + | 0.91 | 1 | acATCTaactt |
| P$LREM | P$RAP22.01 | 0.85 | 790 | 800 | - | 0.902 | 1 | taATCTataat |
| P$LREM | P$RAP22.01 | 0.85 | 931 | 941 | - | 0.921 | 1 | acATCTaagtc |
| P$MADS | P$AG.01 | 0.8 | 15 | 35 | - | 0.813 | 1 | taaTACCtaattagtttggtc |
| P$MADS | P$AGL3.02 | 0.8 | 148 | 168 | - | 0.804 | 0.868 | atttaCCACttttggggtaag |
| P$MADS | P$AGL2.01 | 0.82 | 149 | 169 | + | 0.914 | 0.969 | ttaccCCAAaagtggtaaatt |
| P$MADS | P$AG.01 | 0.8 | 407 | 427 | - | 0.914 | 1 | attTACCttatttggagatat |
| P$MADS | P$SEP3.01 | 0.86 | 408 | 428 | + | 0.915 | 1 | tatctCCAAataaggtaaatt |
| P$MADS | P$AGL15.02 | 0.8 | 453 | 473 | - | 0.835 | 0.858 | attttccctgttaaGTAAgat |
| P$MADS | P$AGL15.01 | 0.79 | 454 | 474 | + | 0.894 | 1 | tctTACTtaacagggaaaatt |
| P$MADS | P$RIN.01 | 0.77 | 623 | 643 | - | 0.778 | 1 | tttagtgagaaATAGcaaaaa |
| P$MIIG | P$PALBOXL.01 | 0.8 | 573 | 587 | + | 0.84 | 1 | ggaagttaGGTGaaa |
| P$MIIG | P$NTLIM1.01 | 0.88 | 995 | 1009 | - | 0.922 | 1 | ttcgggtgtgGTGGg |
| P$MSAE | P$MSA.01 | 0.8 | 961 | 975 | - | 0.899 | 1 | aagcaAACGgaaagt |
| P$MYBL | P$MYBPH3.02 | 0.76 | 14 | 30 | - | 0.78 | 1 | cctaatTAGTttggtcc |
| P$MYBL | P$MYB96.01 | 0.9 | 50 | 66 | + | 0.915 | 1 | attttgaAGTTattatt |
| P$MYBL | P$MYB96.01 | 0.9 | 86 | 102 | + | 0.924 | 1 | aaatactAGTTaactta |
| P$MYBL | P$MYBPH3.02 | 0.76 | 96 | 112 | - | 0.782 | 0.779 | tgcattTCGTtaagtta |
| P$MYBL | P$MYB96.01 | 0.9 | 222 | 238 | + | 0.992 | 1 | attctatAGTTattaat |
| P$MYBL | P$MYB96.01 | 0.9 | 267 | 283 | - | 0.93 | 1 | ctattatAGTTtaaatt |
| P$MYBL | P$MYBPH3.01 | 0.8 | 273 | 289 | - | 0.804 | 0.75 | aataaactATTAtagtt |
| P$MYBL | P$MYBPH3.01 | 0.8 | 284 | 300 | - | 0.801 | 0.75 | cttaaaccTTTAataaa |
| P$MYBL | P$MYBPH3.02 | 0.76 | 323 | 339 | - | 0.782 | 1 | tttagtTAGTatacact |
| P$MYBL | P$MYB96.01 | 0.9 | 327 | 343 | - | 0.976 | 1 | gtgatttAGTTagtata |
| P$MYBL | P$AS1_AS2_I.01 | 0.99 | 456 | 472 | - | 1 | 1 | ttttccCTGTtaagtaa |
| P$MYBL | P$NTMYBAS1.01 | 0.96 | 569 | 585 | + | 0.98 | 1 | agagggaaGTTAggtga |
| P$MYBL | P$MYB96.01 | 0.9 | 603 | 619 | + | 0.904 | 1 | gaaccctAGTTaagggg |
| P$MYBL | P$NTMYBAS1.01 | 0.96 | 857 | 873 | + | 0.962 | 1 | agagagggGTTAgcgtt |
| P$MYBL | P$CARE.01 | 0.83 | 977 | 993 | - | 0.839 | 1 | ttttgggAGTTctgaga |
| P$MYBL | P$MYBPH3.01 | 0.8 | 1006 | 1022 | + | 0.801 | 1 | cgaaaaaaGTTAgagag |
| P$MYBS | P$PHR1.02 | 0.82 | 322 | 338 | + | 0.846 | 0.75 | aagtGTATactaactaa |
| P$MYBS | P$HVMCB1.01 | 0.93 | 342 | 358 | + | 0.976 | 1 | actcctATCCtctcaca |
| P$MYBS | P$TAMYB80.01 | 0.83 | 525 | 541 | - | 0.98 | 1 | ctgcATATtccaacctt |
| P$MYBS | P$PHR1.01 | 0.84 | 530 | 546 | + | 1 | 1 | tggaATATgcaggaaaa |
| P$MYBS | P$MYBST1.01 | 0.9 | 876 | 892 | - | 0.955 | 1 | cattttATCCacctcct |
| P$NACF | P$TANAC69.02 | 0.69 | 87 | 113 | + | 0.708 | 0.803 | aatactagttaacttaACGAaatgcac |
| P$NACF | P$ORS1.01 | 0.65 | 965 | 991 | - | 0.676 | 0.75 | ttgggagttctgagaCAAGcaaacgga |
| P$NCS1 | P$NCS1.01 | 0.85 | 122 | 132 | + | 0.853 | 0.878 | aAAATgtttac |
| P$NCS1 | P$NCS1.01 | 0.85 | 522 | 532 | + | 0.852 | 1 | gAAAAggttgg |
| P$OCSE | P$OCSL.01 | 0.69 | 25 | 45 | - | 0.738 | 0.769 | ttgtgtaatataatACCTaat |
| P$OCSE | P$OCSL.01 | 0.69 | 71 | 91 | - | 0.699 | 0.808 | gtatttaatctcgtACATtta |
| P$OCSE | P$OCSL.01 | 0.69 | 84 | 104 | + | 0.693 | 0.769 | ttaaatactagttaACTTaac |
| P$OCSE | P$OCSL.01 | 0.69 | 319 | 339 | - | 0.709 | 0.769 | tttagttagtatacACTTcta |
| P$OCSE | P$OCSL.01 | 0.69 | 706 | 726 | - | 0.693 | 0.808 | gatagtgatggattACGAccc |
| P$OPAQ | P$O2.02 | 0.87 | 872 | 888 | - | 0.901 | 1 | ttatCCACctcctttaa |
| P$PSPE | P$TL1.01 | 0.91 | 678 | 692 | + | 0.972 | 1 | agtGAAGaaaaaaaa |
| P$PSRE | P$GAAA.01 | 0.83 | 175 | 191 | + | 0.933 | 1 | tgagaGAAAtatttact |
| P$PSRE | P$GAAA.01 | 0.83 | 428 | 444 | + | 0.871 | 1 | ttggaGAAAttttatgc |
| P$ROOT | P$RHE.02 | 0.77 | 121 | 145 | + | 0.781 | 1 | taaaatgtttacgatCACGgcctaa |
| P$SALT | P$ALFIN1.02 | 0.95 | 873 | 887 | + | 0.976 | 1 | taaaggaGGTGgata |
| P$SBPD | P$SPL14.01 | 0.83 | 68 | 84 | - | 0.887 | 1 | atctCGTAcatttatta |
| P$SLIM | P$UPE-BOX.01 | 0.65 | 1054 | 1074 | + | 0.711 | 1 | acaaaCATTgcatttagactc |
| P$SPF1 | P$SP8BF.01 | 0.87 | 366 | 378 | + | 0.877 | 1 | taTACTctttcca |
| P$SPF1 | P$SP8BF.01 | 0.87 | 686 | 698 | - | 0.873 | 1 | tgTACTttttttt |
| P$STKM | P$STK.01 | 0.85 | 276 | 290 | - | 0.871 | 1 | taaTAAActattata |
| P$SUCB | P$SUCROSE.01 | 0.81 | 51 | 69 | - | 0.812 | 1 | taAAATaataacttcaaaa |
| P$SUCB | P$SUCROSE.01 | 0.81 | 799 | 817 | + | 0.816 | 1 | taAAATgagaaaataaaat |
| P$SUCB | P$SUCROSE.01 | 0.81 | 928 | 946 | - | 0.81 | 0.75 | ggAAAAcatctaagtcaac |
| P$SWNS | P$SNBE.01 | 0.92 | 455 | 473 | - | 0.924 | 0.907 | attttccctgtTAAGtaag |
| P$SWNS | P$SNBE.01 | 0.92 | 479 | 497 | - | 0.935 | 1 | aatttagtgagAAAGtaaa |
| P$TEFB | P$TEF1.01 | 0.76 | 132 | 152 | - | 0.77 | 0.957 | gtAAGGgttaggccgtgatcg |
| P$TELO | P$ATPURA.01 | 0.85 | 601 | 615 | + | 0.906 | 1 | cagaACCCtagttaa |
| P$TERE | P$TERE.01 | 0.79 | 867 | 877 | - | 0.911 | 0.806 | ctttAACGcta |
| P$URNA | P$USE.01 | 0.75 | 348 | 364 | + | 0.751 | 0.75 | atcctcTCACatctaac |
| P$WBXF | P$WRKY11.01 | 0.94 | 504 | 520 | + | 0.953 | 1 | tattaTTGActggaaga |

**Table S4. Identification of GV repeats in representative organisms.**

|  | Representative species | Total accessions | Representative annotated proteins | | | Others |
| --- | --- | --- | --- | --- | --- | --- |
|  |  |  | Transcriptional factor | Enzyme | Membrane protein |  |
| Bacteria | *Escherichia coli* | - | - | - | - | - |
|  | *Bacillus subtilis* | - | - | - | - | - |
|  | *Acinetobacter* | 6 |  |  |  | 6 |
|  | *Agrobacterium tumefaciens* | 2 | - | - | - | 2 |
|  | *Amycolatopsis balhimycina* | 1 | - | WP_020640892 | - | -- |
|  | *Amycolatopsis mediterranei* U32 | 1 | - | - | - | 1 |
|  | *Austwickia chelonae* | 1 | - | - | - | 1 |
|  | *Amycolatopsis nigrescens* | 1 | - | - | - | 1 |
|  | *Bradyrhizobium oligotrophicum* S58 | 1 | - | - | - | 1 |
|  | *Brevibacterium massiliense* | 1 | - | - | - | 1 |
|  | *Burkholderia mallei* | 5 | - | - | - | 5 |
|  | *Burkholderia oklahomensis* | 1 | - | - | - | 1 |
|  | *Burkholderia pseudomallei* | 11 | - | - | - | 11 |
|  | *Calothrix* sp. PCC 7103 | 1 | - | - | - | 1 |
|  | *Candidatus Regiella insecticola* | 1 | - | - | - | 1 |
|  | *Capnocytophaga* sp. oral taxon 324 | 1 | - | - | - | 1 |
|  | *Chelatococcus* sp. GW1 | 1 | - | - | - | 1 |
|  | *Corynebacterium matruchotii* | 1 | - | - | - | 1 |
|  | *Frankia* sp CN3 | 5 | - | - | - | 5 |
|  | *Geitlerinema* sp. PCC 7105 | 1 |  |  |  | 1 |
|  | *Gluconacetobacter hansenii* | 1 | - | - | - | 1 |
|  | *Gluconobacter frateurii* | 1 | - | - | - | 1 |
|  | *Gordonia kroppenstedtii* | 1 | - | - | - | 1 |
|  | *Haliangium ochraceum* DSM 14365 | 1 | - | - | - | 1 |
|  | *Kordia algicida* | 1 | - | - | - | 1 |
|  | *Ktedonobacter racemifer* | 1 | - | - | - | 1 |
|  | *marine actinobacterium* PHSC20C1 | 1 | - | - | - | 1 |
|  | *Meganema perideroedes* | 1 | - | - | - | 1 |
|  | *Mesorhizobium amorphae* | 1 | - | - | - | 1 |
|  | *Mesorhizobium* sp. STM 4661 | 1 | - | - | - | 1 |
|  | *Micromonospora* sp. CNB394 | 1 | - | - | - | 1 |
|  | *Mycobacterium tuberculosis* RGTB327 | 1 | - | - | - | 1 |
|  | *Nocardia brasiliensis* ATCC 700358 | 1 | - | - | - | 1 |
|  | *Myxococcus xanthus* DK 1622 | 1 | - | - | - | 1 |
|  | *Nodosilinea nodulosa* | 1 | - | - | - | 1 |
|  | *Novosphingobium* sp. Rr 2-17 | 1 | - | - | - | 1 |
|  | *Plautia stali symbiont* | 1 | - | - | - | 1 |
|  | *Promicromonospora sukumoe* | 1 | - | - | - | 1 |
|  | *Propionibacterium acnes* | 4 | - | - | - | 1 |
|  | *Prosthecochloris aestuarii* DSM 271 | 1 | - | - | - | 1 |
|  | *Rhizobium* *oligotrophicum* S58 | 2 | - | - | - | 2 |
|  | *Rhodococcus* sp. P14 | 1 | - | - | -- | 1 |
|  | *Roseibium* sp. TrichSKD4 | 1 | - | - | - | 1 |
|  | *Rubrivivax benzoatilyticus* | 1 | - | - | - | 1 |
|  | *Saccharothrix espanaensis* DSM 44229 | 1 | - | - | - | 1 |
|  | *Salinispora pacifica* | 3 | - | - | - | 3 |
|  | *Streptomyce* | 16 | WP_009713539  WP_007501539 |  |  | 14 |
|  | *Verrucomicrobia bacterium* | 2 | - | - | - | 2 |
|  | *Salinispora tropica* | 1 | - | - | - | 1 |
| Fungi | *Saccharomyces cerevisiae* | - | - | - | - | - |
|  | *Schizosaccharomyces pombe* | - | - | - | - |  |
|  | *Ajellomyces dermatitidis* | 2 | - | - | - | 2 |
|  | *Arthroderma gypseum* | 2 | - | - | - | 2 |
|  | *Aspergillus clavatus* | 3 | - | - | - | 3 |
|  | *Chaetomium globosum* | 4 | - | - | - | 4 |
|  | *Coprinopsis cinerea* | 4 | - | - | - | 4 |
| Animals | *Caenorhabditis elegans* | - | - | - | - |  |
|  | *Drosophila melanogaster* | 91 | NP_620473  NP_001259125 | NP_726352  NP_001188939  NP_001260984  NP_001259221  NP_001138155  NP_570073  NP_996343  NP_001259220  NP_001138152  NP_525074  NP_001096873  NP_001138153 |  | 77 |
|  | *Danio rerio* | - | - | - | - | - |
|  | *Xenopus* | 1 | - | - | - | 1 |
|  | *Gallus gallus* | 5 | - | XP_418246  XP_004948968 | - | 3 |
|  | *Mus musculus* | 3 | NP_033259 | - | - | 2 |
|  | *Papio anubis* | 3 | - | - | - | 3 |
|  | *Pongo* | 3 | XP_002824485 | - | - | 2 |
|  | *Homo sapiens* | 4 | NP_005977 | - | - | 3 |
| Plants | *Chlamydomonas reinhardtii* | 23 | - | XP_001699076 XP_001690742 | - | 21 |
|  | *Physcomitrella patens* | 3 | - | - | - | 3 |
|  | *Asplenium* | - | - | - | - |  |
|  | *Arabidopsis thiliana* | 7 | NP_192590  NP_564824  NP_001117544  NP_849842  NP_563628 | - | - | 2 |
|  | *Physcomitrella patens* | 6 | - | - | - | 6 |
|  | *Glycine max* | 18 | - | - | - | 18 |
|  | *Zea mays* | 15 | - | - | - | 12 |
|  | *Oryza sativa* | 14 | - | - | - | 14 |
